# Stromal architecture and fibroblast subpopulations with opposing effects on outcomes in hepatocellular carcinoma

**DOI:** 10.1038/s41421-024-00747-z

**Published:** 2025-01-28

**Authors:** Yifei Cheng, Xiaofang Chen, Li Feng, Zhicheng Yang, Liyun Xiao, Bin Xiang, Xiaodong Wang, Dongbin Liu, Penghui Lin, Jieyi Shi, Guohe Song, Wulei Qian, Boan Zhang, Yanan Xu, Zheng Gao, Lv Chen, Yingcheng Wu, Jiaqiang Ma, Youpei Lin, Haichao Zhao, Lihua Peng, Xuebin Mao, Yang Liu, Hao Hou, Mingyu Yang, Yuan Ji, Xiaoying Wang, Jian Zhou, Xun Xu, Xiyang Liu, Wu Wei, Xiaoming Zhang, Qiang Gao, Hu Zhou, Yidi Sun, Kui Wu, Jia Fan

**Affiliations:** 1 Department of Liver Surgery and Transplantation, and Key Laboratory of Carcinogenesis and Cancer Invasion (Ministry of Education), Liver Cancer Institute, Zhongshan Hospital, Fudan University, Shanghai, China; 2 HIM-BGI Omics Center, Zhejiang Cancer Hospital, Hangzhou Institute of Medicine (HIM), Chinese Academy of Sciences (CAS), BGI Research, Hangzhou, Zhejiang China; 3 Guangdong Provincial Key Laboratory of Human Disease Genomics, BGI Research, Shenzhen, Guangdong China; 4 College of Life Sciences, University of Chinese Academy of Sciences, Beijing, China; 5 Institute of Neuroscience, State Key Laboratory of Neuroscience, Key Laboratory of Primate Neurobiology, Center for Excellence in Brain Science and Intelligence Technology, Chinese Academy of Sciences, Shanghai, China; 6 Department of Analytical Chemistry, State Key Laboratory of Drug Research, Shanghai Institute of Materia Medica, Chinese Academy of Sciences, Shanghai, China; 7 University of Chinese Academy of Sciences, Beijing, China; 8 Key Laboratory of Computational Biology, Shanghai Institute of Nutrition and Health, University of Chinese Academy of Sciences, Shanghai, China; 9 School of Computer Science and Technology, Xidian University, Xi’an, Shaanxi China; 10 BGI Research, Qingdao, Shandong China; 11 Department of Pathology, Zhongshan Hospital, Fudan University, Shanghai, China; 12 Guangdong Provincial Key Laboratory of Genome Read and Write, BGI Research, Shenzhen, Guangdong China; 13 The Center for Microbes, Development and Health, Key Laboratory of Molecular Virology & Immunology, Shanghai Institute of Immunity and Infection, Chinese Academy of Sciences, Shanghai, China; 14 Institute of Intelligent Medical Research (IIMR), BGI Genomics, Shenzhen, Guangdong China

**Keywords:** Cancer microenvironment, Tumour immunology

## Abstract

Dissecting the spatial heterogeneity of cancer-associated fibroblasts (CAFs) is vital for understanding tumor biology and therapeutic design. By combining pathological image analysis with spatial proteomics, we revealed two stromal archetypes in hepatocellular carcinoma (HCC) with different biological functions and extracellular matrix compositions. Using paired single-cell RNA and epigenomic sequencing with Stereo-seq, we revealed two fibroblast subsets CAF-FAP and CAF-C7, whose spatial enrichment strongly correlated with the two stromal archetypes and opposing patient prognosis. We discovered two functional units, one is the intratumor inflammatory hub featured by CAF-FAP plus CD8_PDCD1 proximity and the other is the marginal wound-healing hub with CAF-C7 plus Macrophage_SPP1 co-localization. Inhibiting CAF-FAP combined with anti-PD-1 in orthotopic HCC models led to improved tumor regression than either monotherapy. Collectively, our findings suggest stroma-targeted strategies for HCC based on defined stromal archetypes, raising the concept that CAFs change their transcriptional program and intercellular crosstalk according to the spatial context.

## Introduction

Liver cancer is a highly lethal malignancy that causes around 800,000 deaths annually^1^, with hepatocellular carcinoma (HCC) comprising 75%–85% of the total cases. Approximately 50%–60% of HCC patients were diagnosed as advanced-stage, receiving systemic therapies^2^. However, even the first-line immune checkpoint blockades (ICBs) offer limited efficacy and response duration^3^. Of note, HCC typically arises in a stroma-rich environment, with liver fibrosis existing in 80%‒90% of the patients^4^ secondary to common etiologies, such as chronic viral infection, metabolic syndrome, and alcohol abuse^5^. Intratumor stroma, occupying tumor mass, is associated with T cell exclusion and ICB efficacy in HCC^6^. Considering that fibroblasts are the building blocks of stromal architecture and cancer-associated fibroblasts (CAFs) are actively involved in tumor progression through complex crosstalk with other cell types in the tumor microenvironment (TME)^7^, targeting CAFs may augment the effect of current immunotherapies^8^, which is supported by accumulating evidence from both clinical trials^9^ and pre-clinical models^10,11^. However, CAFs remain therapeutically underexplored in HCC, limited by current knowledge of the biological diversity of CAF. Meanwhile, the fibrotic ring (FR), surrounding tumor mass, also known as tumor encapsulation^12^, was reported to have contradictory prognostic roles in HCC^13,14^. Elucidating the biological function and regulatory mechanisms of spatially different stroma components may inform novel therapeutic options in HCC.

It is long recognized that the type, density, and location of immune cells within distinct tumor subregions dictate patient outcome and treatment efficacy across multiple cancer types^15,16^, including HCC^17^. Recently, fibroblasts have emerged as the organizers of immune compartmentalization in secondary lymphoid organs^18^ and within TME^19^. By single-cell RNA-sequencing (scRNA-seq), several CAF subpopulations^20–22^ or CAF-oriented spatial hubs^23^ have been identified as biomarkers for ICB response in pancreatic, colorectal, and head and neck cancers. Although recent human HCC scRNA-seq datasets captured tumor and non-tumor tissue fibroblasts^6,24–29^, few studies obtained CAFs from distinct tumor sub-regions of HCC, especially the tumor‒liver interface^28^, hampering the detection of the full spectrum of CAF subpopulations with spatial information.

Cutting-edge spatial transcriptomics (ST), capturing tissue transcriptome while preserving spatial coordinates, have been used to uncover fundamental interactions between fibroblasts and other intratumoral components with spatial preference that govern disease progression and therapeutic response^30,31^. However, both scRNA-seq and ST inevitably lose the extracellular matrix (ECM) profiles that affect CAF plasticity and immune cell localization^32^. Combining the emerging high-resolution ST techniques like SpaTial enhanced resolution omics-sequencing (Stereo-seq)^30^ and spatial proteomics^33^ may allow deciphering single-cell spatial landscapes together with ECM compositions and may complement up-to-date understandings of tumor stroma‒immune interactions.

Here, we applied a multimodal approach to investigate the tumor-margin-liver axis of HCC, identifying two distinct stromal archetypes (“FR^+^” with FR surrounding tumor mass and “FR^‒^” harboring prominent intratumor stroma but without FR). Using image deep learning, SpaTial enhanced resolution omics-sequencing (Stereo-seq), single-cell/nuclei RNA-sequencing, and spatial proteomics, we revealed that the two stromal archetypes in HCC had distinct prognostic, immunologic, and ECM profiles. We found that two fibroblast subpopulations (CAF-FAP and CAF-C7) were separately enriched at the intratumor stroma and surrounding FRs, and their spatial balance dictated clinical outcomes. We identified RUNX1 and USF2 as potential regulators for CAF-FAP and CAF-C7 differentiation, respectively, and discovered that they may rewire the TME by organizing pro-inflammatory and wound-healing hubs. Our findings provide new insights into the spatial biology of CAFs in HCC, raising the concept that specific CAF subsets fit their regional transcriptional programs and intercellular crosstalk to the tissue context.

## Results

### Stromal architecture in HCC is associated with patient survival

**Fig. 1:**
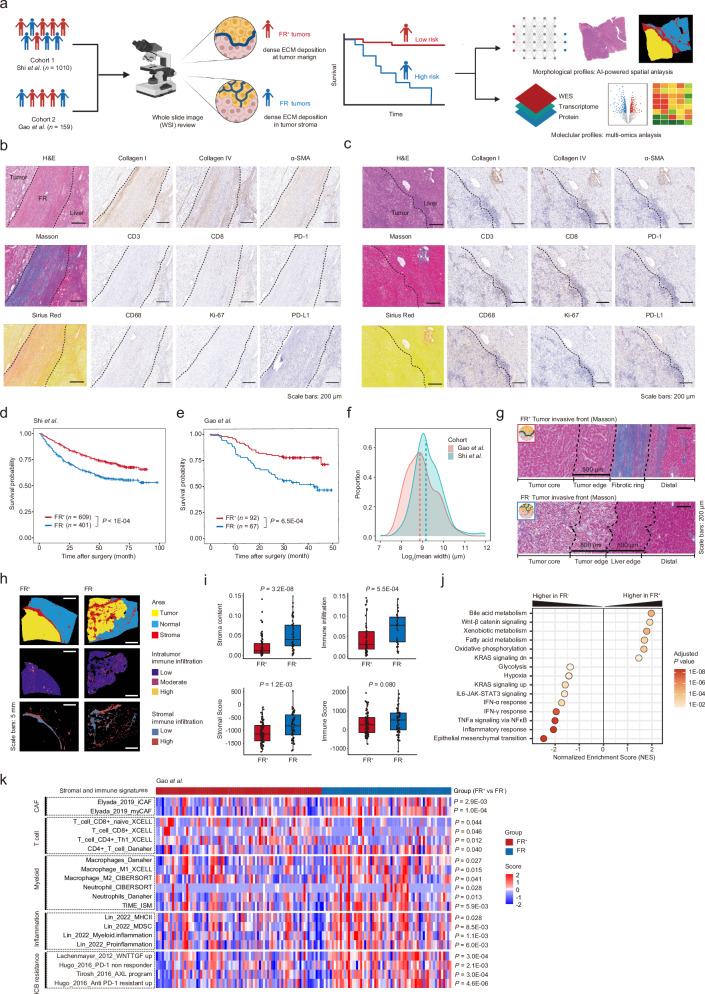
Stromal architecture in HCC and its clinical and multiomic correlations. **a** Schematic diagram of stromal architecture identification, public cohorts, and analytical approaches. **b**, **c** Representative H&E, Masson, Sirius Red, and IHC staining showing collagen fibers (Collagen I, Collagen IV), fibroblast activation (a-SMA), immune infiltration (CD3, CD8, CD68), tumor proliferation (Ki-67), and checkpoint molecules (PD-1, PD-L1) at the tumor margin of FR^+^ tumors (**b**) and FR^‒^ tumors (**c**). Scale bars: 200 μm. **d**, **e** Kaplan-Meier curves for overall survival in cohort 1 (**d**) and cohort 2 (**e**) based on stromal architecture. Log-rank test. **f** The distribution of tumor-surrounding stroma width in cohort 1 and cohort 2. **g** Representative images of FR^+^ tumors (upper) and FR^‒^ tumors (bottom) at the invasive front by Masson trichrome staining, illustrating the definition of 4 spatial zones. **h** Visualization of tissue type (upper), intratumor immune infiltration (middle), and stromal immune infiltration (lower) in representative WSIs of FR^+^ (left) and FR^‒^ (right) tumors predicted by deep learning models. **i** Boxplots comparing the distribution of intratumor stromal content (left) and intratumor immune infiltration (right) predicted by deep learning and RNA-seq in FR^+^ and FR^‒^ tumors. Wilcoxon test. **j** GSEA of the HALLMARK genesets showing ontologies enriched in FR^+^ and FR^‒^ tumors, respectively. A normalized enrichment score is given for each ontology; points are colored by the adjusted *P* value. **k** Comparison of stromal and immune signatures between FR^+^ and FR^‒^ HCC tumor tissue from cohort 2. Wilcoxon test.

To study regional stromal patterns that recurred in HCC patients, we systematically reviewed 1169 treatment-naïve HCC cases from 2 well-documented cohorts (cohort 1, *n* = 1010; cohort 2, *n* = 159)^34,35^ with whole-slide images (WSIs) of hematoxylin and eosin (H&E) stained tissue sections and identified two major stromal archetypes in HCC (Fig. 1a; Supplementary Table S1): (1) Tumors with FRs around the tumor margin (FR^+^ tumors) (2) Tumors with enriched intratumor stroma but without FR (FR^‒^ tumors).

Histologically, FR is featured by the conspicuous presence of ECM deposition at the tumor margin, surrounding the tumor mass, as shown in H&E staining (Fig. 1b, c). Notably, fibers within FR exhibit sheet-like alignment, which distinguishes FR from intratumor or liver stroma that typically less-organized. Furthermore, the FR area exhibited positive staining for Masson and Sirius Red, indicative of abundant collagen deposition. IHC staining confirmed the increased abundance of collagens and fibroblast activation (collagen I, collagen IV, a-SMA) at the FR^+^ tumor margin (Fig. 1b, c) and showed elevated staining of fibroblast activation and immune exhaustion in FR^‒^ tumor stroma (Supplementary Fig. S1a, b), underscoring broad divergence between these two stromal archetypes. Interestingly, we rarely observe FR in other primary liver cancer types like intrahepatic cholangiocarcinoma and liver metastases from various origins (Supplementary Fig. S1c), suggesting that the FR is a unique characteristic of HCC compared to other liver malignancies.

Clinically, patients with FR^+^ tumors had better survival compared to FR^‒^ tumors in both cohort 1 (Fig. 1d; *P* < 1E‒04) and cohort 2 (Fig. 1e; *P* = 6.5E‒04), consistent with previous reports in HCC^13,36,37^. Multivariate Cox analysis confirmed that FR^+^ remained a favorable prognostic feature in both cohorts (Supplementary Fig. S1d). Notably, the prevalence of FR was broadly comparable along small to intermediate and huge size tumors (range of ≤ 1 cm to > 10 cm), suggesting that the stromal archetypes may be imprinted early during tumor initiation and kept through tumor progression (Supplementary Fig. S1e).

Next, using our previously developed deep learning models, we measured the FRs using H&E sections. We revealed that its mean width fit into a normal distribution with a median width of approximately 500 μm (Fig. 1f), comparable to the span of what we previously defined as HCC tumor margin^38^. Based on these observations, we compartmentalized regions on tissue sections into four zones (i.e., tumor core, tumor edge, liver edge, and distal) (Fig. 1g). Specifically, edge regions were defined as extensions of 500 μm from the tumor‒normal borderline. For FR^+^ tumors, the width of liver edge was recorded as the entire width of the FR to preserve inter-patient heterogeneity. Stromal and immune cells exhibited similar increasing trends of intratumor enrichment in FR^‒^ tumors compared to FR^+^ tumors, as assessed by both morphological and transcriptional estimations (Fig. 1h, i; Supplementary Fig. S1f), while in the non-tumor liver, stroma content and immune infiltration showed only slight difference between the FR^‒^ and FR^+^ groups (Supplementary Fig. S1g). In concordance, histological grading of liver fibrosis was comparable in FR^+^ and FR^‒^ HCC patients (Supplementary Fig. S1h). Collectively, non-tumor liver stroma may play a less prominent role in FR maintenance than the tumor itself.

By associating the stromal features with multi-omics data in cohort 2, whole exome sequencing (WES) analysis revealed no significant differences in tumor mutation burden (TMB), tumor neoantigen burden (TNB), or recurrent mutations between FR^‒^ and FR^+^ tumors (Supplementary Table S1). According to previous HCC molecular classification, FR^+^ tumors were enriched for good-prognosis subtypes, such as the metabolic subgroup^34^ (Supplementary Fig. S1i). Differential gene expression (DGE) and gene set enrichment analysis (GSEA) (Fig. 1j; Supplementary Fig. S1j and Table S1) showed that FR^+^ tumor tissue is enriched for pathways related to liver-specific metabolism (xenobiotic metabolism, fatty acid metabolism, and OXPHOS) and WNT/β-catenin signaling while FR^‒^ tumors showed upregulated pathways related to inflammation (IFN-γ pathway, TNF-α pathway), immune suppression (IL-6-JAK-STAT3 signaling), and epithelial-mesenchymal transition (EMT), largely consistent at both mRNA and protein levels. Further comparison of previously published stroma and immune signature expression in FR^+^ and FR^‒^ HCC tumor tissue from cohort 2 (Fig. 1k) revealed that FR^+^ HCCs, despite exhibiting a lower overall Immunescore, displayed a significantly higher proportion of CD8^+^ T cells, particularly CD8^+^ naïve T cells, suggesting a more balanced TME. Conversely, FR^‒^ HCCs, which had a higher Immunescore, were enriched for myeloid cells and exhibited increased inflammation and immunotherapy resistance signatures, aligning with a less favorable prognosis observed in FR^‒^ HCCs (Fig. 1d, e). Consistently, within the ERP117672 cohort, we found non-responders showed higher intratumor stromal scores and fibroblast abundance, while responders were notably enriched for CD8^+^ T cells, especially CD8^+^ naïve T cells (Supplementary Fig. S1k). However, the inflamed class signature^39,40^, IFNAP signature^3^, and inflammatory signature^41^ showed no significant difference between FR^+^ and FR^‒^ tumor tissues in cohort 2 (Supplementary Fig. S1l), which warrant future investigation. This lack of difference may be partly due to that these 3 signatures do not fully account for variations in myeloid inflammation and stromal disturbances, which also potentially influence ICB response.

### Proteomic profiling unveils regional stromal differences of FR^+^ and FR^‒^ tumors

**Fig. 2:**
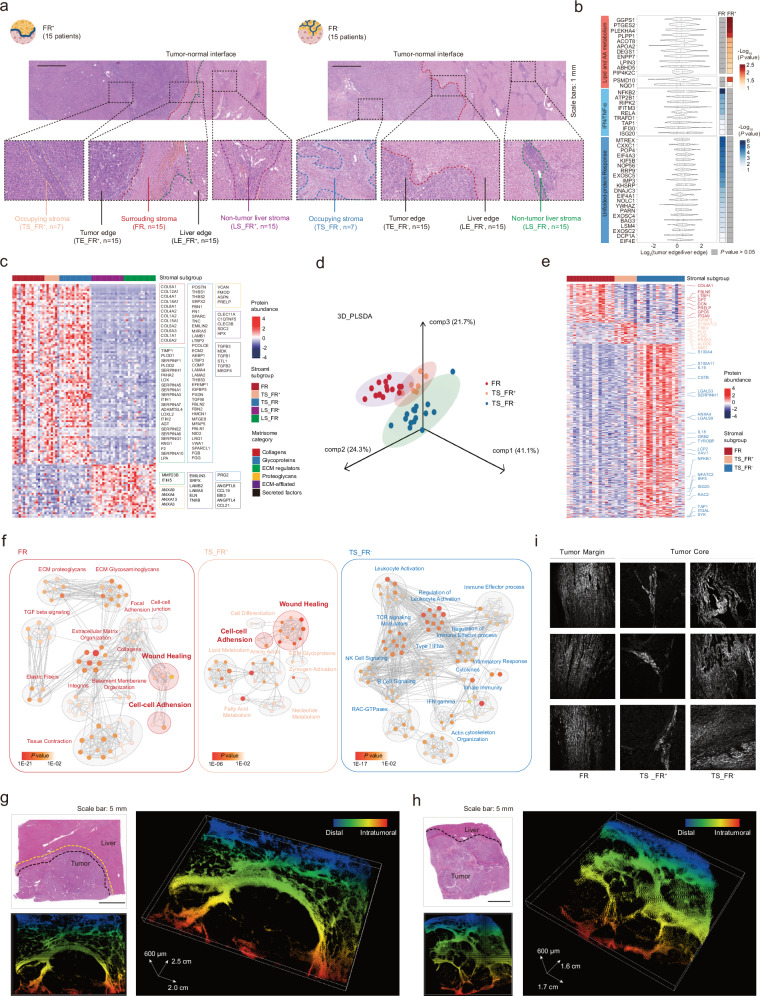
Spatial proteomics profiling of two stromal archetypes in HCC. **a** Schematic diagram of morphology-guided LCM of tumor margin samples of HCC (FR^+^, *n* = 15; FR^‒^, *n* = 15) at TS, FR, LS, TE and LE, followed by proteomic analysis (*n* = 127). Scale bars: 1 mm. TS tumor-occupying stroma, FR fibrotic ring, LS non-tumor liver stroma, TE tumor edge, LE liver edge. **b** Violin plot showing the abundance of proteins related to lipid and amino acid metabolism, inflammation, and stress response in TE compared with LE. Heatmap displays the *P* values of comparisons between TE and LE. Wilcoxon test. **c** Heatmap of the differentially expressed proteins in the cancer-associated stroma (FR, TS_FR^+^, TS_FR^‒^) or non-tumor liver stroma (LS_FR^+^, LS_FR^‒^) (*P* < 0.05). Paired *t*-test. ECM-related proteins were shown and classified by their ECM category. **d** Partial Least Squares Discriminant Analysis (PLS-DA) of FR, TS_FR^+^, TS_FR^‒^. **e** Heatmap of the differentially expressed proteins among cancer-associated stroma (FR, TS_FR^+^, TS_FR^‒^). **f** Network of the genesets upregulated in FR, TS_FR^+^, and TS_FR^‒^, respectively. **g**, **h** H&E (top left) and 3D reconstruction of the stromal architecture in FR^+^ (**g**) and FR^‒^ (**h**) HCC. Scale bars: 5 mm. **i** Representative SHG images from FR (left), TS_FR^+^ (middle), and TS_FR^‒^ (right), respectively.

Regional stromal features, including cellular and acellular ECM components, were crucial for understanding TME spatial organization as they dynamically change depending on the tissue context. However, bulk sequencing analysis may not fully reflect spatial stromal heterogeneities in HCC TME, particularly for tumors with low stromal content. Therefore, we applied a histology-guided spatial proteomics workflow to dissect HCC stroma and investigate their proteomic features. We conducted multi-region sampling of 127 formalin-fixed paraffin-embedded (FFPE) samples from 30 treatment-naïve HCC patients (FR^‒^, *n* = 15; FR^+^, *n* = 15) using laser capture microdissection (LCM) (Fig. 2a; Supplementary Fig. S2a and Table S2). We obtained the paired tumor-occupying stroma (TS, *n* = 22; TS_FR^+^, *n* = 7; TS_FR^‒^, *n* = 15), non-tumor liver stroma (LS, *n* = 30; LS_FR^+^, *n* = 15; LS_FR^‒^, *n* = 15), tumor edge (TE, *n* = 30; TE_FR^+^, *n* = 15; TE_FR^‒^, *n* = 15), and liver edge (LE, *n* = 30; LE_FR^+^, *n* = 15; LE_FR^‒^, *n* = 15) from both FR^+^ and FR^‒^ groups and collected FRs (*n* = 15) from the FR^+^ group. We could only analyze 7 TS_FR^+^ from the 15 FR^+^ cases since the LCM on the other 8 FR^+^ tumor samples generated insufficient material for proteomic experiments due to the scarcity of tumor-occupying stroma (Fig. 1h, i).

Spatial proteomics identified 9167 unique proteins (Supplementary Fig. S2b), capturing a broad spectrum of ECM proteins. Principal component analysis (PCA) based on spatial proteomic profiles revealed two clusters corresponding to dissected stromal and epithelial components in both tumor and liver tissues (Supplementary Fig. S2c, d and Table S2), and the expression profiles of stromal components (FR, TS, and LS) were well-aligned with established stromal signatures^42^ (Supplementary Fig. S2e). For the malignant epithelial, functional enrichment showed upregulated metabolic pathways in TE_FR^+^, while inflammation pathways were elevated in TE_FR^‒^ (Fig. 2b; Supplementary Table S2). This result largely coincides with bulk tumor profiles (Fig. 1j; Supplementary Fig. S1j). Interestingly, we discovered higher activation of unfolded protein response (UPR) pathway in TE_FR^‒^ than TE_FR^+^ (Fig. 2b) or LE_FR^‒^ (Supplementary Fig. S2f, g), which is closely related to the stress response, indicating a potential mechanism of local inflammation in FR^‒^ tumors. Accordingly, we observed increased expression of canonical stress-related and inflammatory proteins (SAA, CRP, HSP90, MMP14, and IL-8) in FR^‒^ HCCs by IHC staining (Supplementary Fig. S2h, i). SAA was reported to alter the liver’s immune and fibrotic microenvironment to an inflammatory phenotype in patients with liver metastasis and intrahepatic cholangiocarcinoma^43,44^, implying SAA may be a universal pro-inflammatory indicator across liver malignancies.

For stromal components, we observed upregulation of collagens and glycoproteins in TS and FR compared with LS (Fig. 2c; Supplementary Table S2) in accordance with previously reported cancer-associated matrix proteins^45^. In addition to shared proteins, partial least squares discriminant analysis (PLS-DA) identified unique protein profiles of FR, TS_FR^+^, and TS_FR^‒^ (Fig. 2d; Supplementary Table S2). FR exhibited a high level of basement membrane proteins and fibroblast lineage markers (COL4A1, FBLN5, DCN, DPT) and is enriched for ECM organization, wound-healing, and cell adhension pathways (Fig. 2e, f), suggesting a potential shared mechanism between tumor-surrounding stroma and tissue repair. TS_FR^+^ was characterized by enrichment of metabolic enzymes and ECM proteoglycan (ALDOC, KMO, TSKU) and featured pathways of lipid, amino acid, and nucleotide metabolism (Fig. 2e, f), consistent with the upregulation of various metabolic pathways in FR^+^ tumor epithelial, indicating a uniform metabolic preference for stroma and the tumor they reside in. TS_FR^‒^ showed upregulated immune-regulatory and ECM degradation proteins (IL-18, LGALS9, ISG20, and CTSB), along with enriched pathways related to actin cytoskeleton organization, RAC-GTPases, and leukocyte activation, in parallel with FR^‒^ tumor profiles (Fig. 2e, f), indicating that dysregulated fibroblasts and immune cells may collaborate within the dense stroma in FR^‒^ tumors.

Interestingly, TS_FR^+^ shared upregulation of cell‒cell adhesion and wound-healing process with FR (Fig. 2e, f), suggesting a functional relevance between spatially linked stromal components. Concordantly, FR ranked highest in wound-healing signature expression across 5 stroma regions, followed by TS_FR^+^ (Supplementary Fig. S2j). Three-dimensional stromal reconstruction confirmed the broad interconnections of the stroma from different sublocations in both FR^+^ and FR^‒^ tumors (Fig. 2g, h). Notably, both the TS_FR^‒^ and FR were linked with the LS, suggesting that TS_FR^‒^ and FR may originate from the non-tumor LS during tumor progression but later deviate due to different spatial clues. Second-harmonic generation (SHG) further revealed the delicate morphology of collagen fibers (Fig. 2i), the main structural element of ECM, among FR, TS_FR^+^, and TS_FR^‒^. Collagen fibers with a distinct sheet-like structure lined the tumor edge of FR^+^ tumors, which may function as biological barriers between the tumor and adjacent normal tissue to inhibit inflammatory crosstalk and tumor invasiveness at the front. At the tumor core, TS_FR^‒^ demonstrated dense, cross-linked collagen fibers under SHG, contrary to thinner and less aggregated fibers in TS_FR^+^. The altered ECM fibers around the tumor mass may correlate with decreased T cell motility^46^ and restricted T cell migration^47^, according to prior studies in human lung cancer. These distinct molecular features revealed by spatial proteomics indicate fundamental spatial heterogeneities in the stromal architecture of FR^+^ and FR^‒^ tumors.

### ST reveals fibroblasts contributing to spatial stromal heterogeneity

**Fig. 3:**
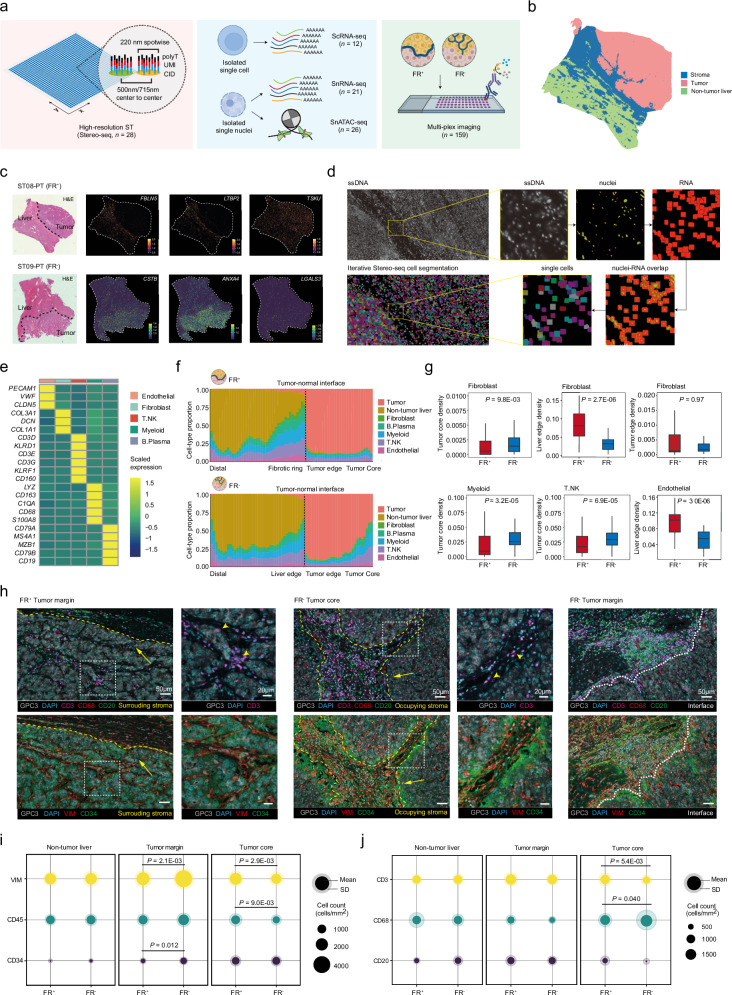
Deciphering HCC spatial stromal heterogeneity at single-cell level. **a** Schematic overview of the spatial multi-OMIC workflow, including Stereo-seq, scRNA-seq, snRNA-seq, snATAC-seq, and multi-plex imaging. **b** Spatial mapping of the 3 major regions based on spatial confined clustering. **c** Paired H&E images and spatial visualization of representative stromal gene expression of FR, TS_FR^+^, and TS_FR^‒^. **d** Illustration of the single-cell segmentation workflow. Nuclei were measured by ssDNA staining, and cell boundaries were determined by captured RNA signals. **e** Heatmap showing the marker gene expression of the 5 major clusters by single-cell ST. **f** Stacked plot showing the distribution of all major cell types by layer along the tumor-margin-liver axis in FR^+^ tumors (upper) and FR^‒^ tumors (lower). **g** Boxplot comparing the spatial enrichment of 4 major clusters in defined spatial zones. Wilcoxon test. **h** Representative multi-plex immunostaining images of fibroblast (VIM^+^), T cell (CD3^+^), macrophage (CD68^+^), B cell (CD20^+^), and endothelial (CD34^+^) marker expression at the invasive boundary of FR^+^ tumors (left) and FR^‒^ tumors (right). **i**, **j** Comparisons of major TME cell distribution between FR^+^ tumors and FR^‒^ tumors across different spatial regions. Circle plots represent the mean and standard deviation (SD) of the cell densities (cells/mm^2^) in TMAs of paired non-tumor, tumor margin, and tumor core from cohort 2 (*n* = 159; FR^+^, *n* = 92; FR^‒^, *n* = 67). The statistics of fibroblasts (VIM^+^), endothelial cells (CD34^+^), and immune cells (CD45^+^) were included in (**i**), while T cells (CD3^+^), macrophages (CD68^+^), and B cells (CD20^+^) were displayed in (**j**). Wilcoxon test.

We next generated a single-cell spatial atlas of 28 HCC samples (patients *n* = 28; FR^+^, *n* = 18; FR^‒^, *n* = 10) to study the cellular components that mainly contribute to the regional stromal features. All data were obtained from matched patient samples and analyzed using Stereo-seq (*n* = 28), complemented by single-nucleus RNA sequencing (snRNA-seq, *n* = 21), scRNA-seq (*n* = 12), and single-nucleus assay for transposase-accessible chromatin using sequencing (snATAC-seq, *n* = 26) (Fig. 3a; Supplementary Fig. S3a and Table S3).

Paired WES data of samples that had undergone spatial sequencing confirmed no significant difference in TMB and TNB between FR^+^ and FR^‒^ tumors (Supplementary Table S3), aligning with the observations in cohort 2 (Supplementary Table S1). Stereo-seq generated 2,091,321 valid bin50 observations (bin50, equivalent to around 25 μm × 25 μm) with an average capture of 1020 genes per bin50 (Supplementary Table S3). Spatially confined clustering revealed three major regions (i.e., tumor, non-tumor liver, and stroma) featured by known marker genes, respectively (Fig. 3b; Supplementary Fig. S3b and Table S3). Consistent with spatially confined ECM proteins identified by spatial proteomics (Fig. 2e), we also found *FBLN5* and *LTBP1* enriched in FR, *TSKU* enriched in TS_FR^+^, and *CSTB*, *ANXA4*, *LGSAL3* enriched in TS_FR^‒^ (Fig. 3c), suggesting coherent activation of ECM remodeling in HCC stromal architecture at both RNA and proteomic levels.

We then developed a single-cell segmentation algorithm, Iterative Stereo-seq, to accurately segment single cells on the Stereo-seq map (Fig. 3d; Supplementary Fig. S3c, d and Table S3), and the segmented single cells were annotated based on scRNA-seq identified cell types (Fig. 3e; Supplementary Fig. S3e, f). Using the cell-type resolved spatial maps, we further studied the spatial gradients of stromal compositions along the tumor-margin-liver axis (Fig. 3f). Unsurprisingly, tumor cells were the densest population in the tumor core and tumor edge, and hepatocytes were enriched in the distal zone. Stromal and immune cell components were mostly enriched within the 500 μm edge regions (tumor edge and liver edge) (Supplementary Fig. S3i). Specifically, increased fibroblasts were detected at the FR in FR^+^ tumors, and FR^‒^ tumors harbored more fibroblasts in tumor core, with no significant differences in the tumor edge between FR^+^ and FR^‒^ tumors (Fig. 3g). The uneven fibroblast distribution aligns with the profound spatial, functional, and morphological differences between FR and TS_FR^‒^, highlighting the critical role of fibroblast heterogeneity in shaping HCC stromal architecture. In addition to fibroblasts, total myeloid and T cells were higher in FR^‒^ HCC tumor core (Fig. 3g), in agreement with higher inflammation in TS_FR^‒^ (Fig. 2f). Moreover, endothelial cells co-localized with fibroblasts at FR may help maintain ECM deposition programs, as suggested by previous reports on their similar involvement in liver fibrosis^48^.

Multiplex IHC showed that T cells were restricted in the tumor stroma of FR^‒^ tumors (Fig. 3h), in combination with elevated tumor WNT-TGF and AXL programs by bulk RNA-seq analysis (Fig. 1k), supporting that cross-linking fibers may inhibit T cell infiltration in FR^‒^ tumors (Fig. 2i). Meanwhile, IHC displayed enrichment of immune cells at the tumor margin of FR^‒^ tumors (Fig. 3h). Then, IHC validation on tissue microarrays (TMAs) from cohort 2 (*n* = 159, Fig. 3i, j; Supplementary Fig. S3j, k and Table S3) confirmed the spatial distribution of TME cells according to Stereo-seq analysis except for the enrichment of endothelial cells at tumor core (Fig. 3g). Misclassification of endothelial cells may be due to the diffusion effects of current sequencing-based ST, making it challenging to precisely distinguish flattened endothelial cells from the surrounding tumor mass. Thus, we excluded them from subtype mapping in the following analysis. Given the spatial enrichment of fibroblasts at the tumor-surrounding and tumor-occupying stroma, we hypothesized that fibroblast spatial heterogeneity may shape local stromal architecture, thus influencing tumor biology and immune milieu.

### CAF-FAP and CAF-C7 are associated with two stromal archetypes

Although diverse CAF subsets have been reported by previous single-cell characterization, their pro-tumor and anti-tumor roles varied among different cancer types^7^, and the spatial complexity of different fibroblast subpopulations remain poorly described in HCC^24,49^. Given that the fibroblast composition was profoundly altered according to the spatial positioning of HCC stroma, we analyzed snRNA-seq data of cells derived from spatial-sequenced samples to reveal the spatial-functional relevance of fibroblasts. We characterized the fibroblast cluster by expression of known marker genes (*COL1A1*, *COL1A2*) and lack of expression of epithelial, immune, and endothelial genes (*GPC3*, *HAMP*, *PTPRC*, and *CLDN5*), yielding a total of 6828 cells.

**Fig. 4:**
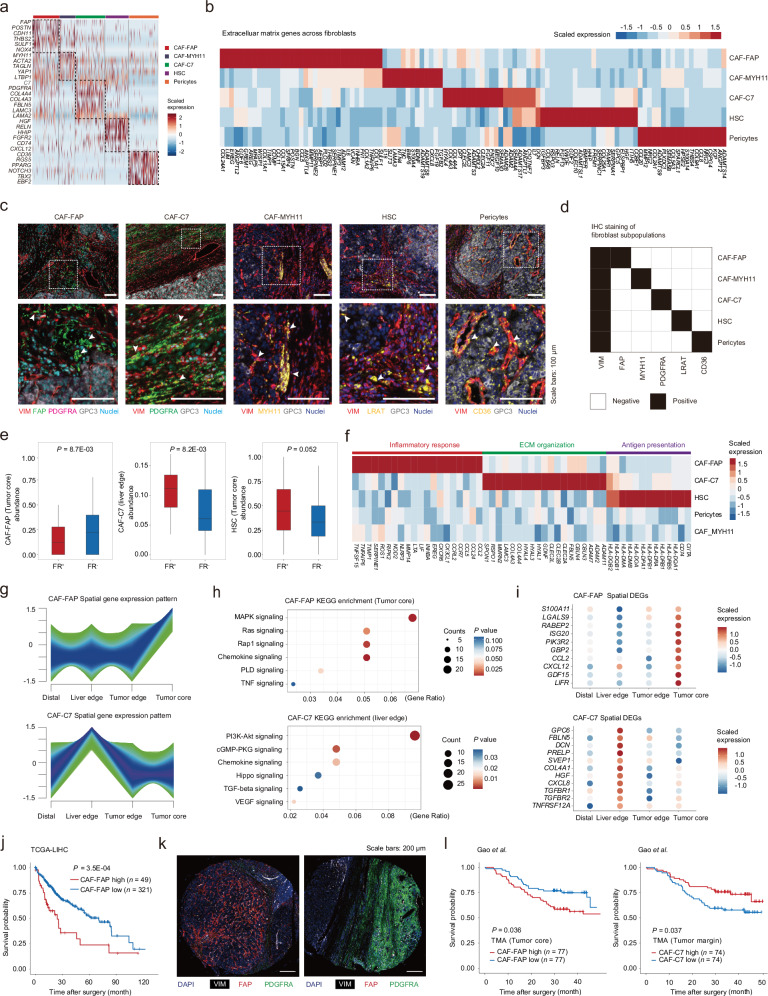
Distinct CAF subpopulations associated with stromal archetypes. **a** Heatmap showing the expression profiles of fibroblast subsets by snRNA-seq. **b** Average gene expression of highly variable ECM genes in fibroblast subclusters by snRNA-seq. **c** Representative images of multi-plex imaging for fibroblast subtyping markers identified by snRNA-seq. Arrows highlight cells of interest. Scale bars: 100 μm. **d** Presentation of the 5 subtyping markers on the identified fibroblast clusters. **e** Boxplot comparing the spatial enrichment of fibroblast subsets in defined spatial zones (FR/liver edge and tumor core) in FR^+^ tumors vs FR^‒^ tumors. Student’s *t*-test. **f** Average gene expression of featured functional genes (related to inflammatory response, ECM organization, antigen presentation) in each fibroblast subset by snRNA-seq. **g** The gene expression patterns along the tumor-margin-liver axis of CAF-FAP (upper) and CAF-C7 (lower) by Stereo-seq. **h** KEGG enrichment of spatial DEGs present in the pattern of CAF-FAP (upper) and CAF-C7 (lower). **i** Dot plot showing the expression of the representative genes involved in chemokine signaling in CAF-FAP (upper) and CAF-C7 (lower), which were also detected by spatial proteomics. **j** Kaplan-Meier curves for overall survival in TCGA-LIHC cohort (*n* = 370) based on CAF-FAP signature. Log-rank test. **k** Representative multi-plex images of CAF-FAP (VIM^+^FAP^+^) at tumor core (FR^‒^) and CAF-C7 (VIM^+^PDGFRA^+^) at tumor margin (FR^+^). Scale bars: 200 μm. **l** Survival analysis based on immunostaining scores of CAF-FAP at tumor core (left, *n* = 154) and CAF-C7 at tumor margin (*n* = 148, right). Log-rank test.

Re-clustering of the defined fibroblasts identified five subpopulations (Fig. 4a; Supplementary Fig. S4a and Table S4), reflecting fibroblast heterogeneity in HCC. Fibroblasts were annotated based on their unique marker gene profiles. CAF-FAP displayed enriched expression of *FAP*^47^, *POSTN*, *THBS2*, and *NOX4*, while CAF-MYH11 exhibited high levels of genes involved in canonical contraction, notably *MYH11*^47^ and *TAGLN*. CAF-C7 was characterized by elevated expression of *C7* and *PDGFRA*, where *C7* is associated with complement activation in CAFs^50^ and *PDGFRA* marks scar-associated mesenchymal cells in liver fibrosis^51^. Hepatic stellate cells (HSCs) were identified by *HGF* and *RELN*, with *RELN* serving as a marker for HSCs^52^ and *HGF* associated with cytokine- and growth-factor-expressing HSCs (cyHSCs)^53^. Notably, *CD74* and *CXCL12* were also upregulated in HSCs, suggesting a potential role in antigen presentation. Pericytes showed distinct expression patterns of canonical markers like *PDGFRB*, *RGS5*, *CSPG4*, and *MCAM*^54,55^, with minimal expression of endothelial markers (*PECAM1* and *CD34*) (Supplementary Fig. S4b). Furthermore, the F8^+^ CAF signature identified by Li et al.^56^ in HCC and FAP^+^ CAF signature derived from human lung cancer^47^ were both enriched in CAF-FAP (Supplementary Fig. S4c). ECM gene expression profiles also distinguished the above fibroblast clusters (Fig. 4b), suggesting distinct ECM proteins may be closely associated with a given fibroblast state. We further validated the existence of the above fibroblast subtypes from human HCC in mouse hydrodynamic tail vein injection (HDTVi) models (Supplementary Fig. S4d, e). Several subclusters, including CAF-FAP, were shared between the murine HCC models and their human counterparts. Multi-plex IHC (Fig. 4c, d) and Stereo-seq deconvolution (Supplementary Fig. S4f) in human HCC tissue also confirmed distinct spatial patterns of fibroblast subsets.

We then combined snRNA-seq, Stereo-seq, and multi-plex imaging to study the spatial positioning and biological function of the fibroblast subpopulations. We assumed that fibroblast subsets enriched at the tumor core and tumor margin were more likely the choreographers of regional stromal architecture. Thus, we mapped the five fibroblast subsets onto the Stereo-seq data (Supplementary Fig. S4g and Table S4). Spatially, we identified CAF-FAP and CAF-C7 as the potential organizers of intratumoral and marginal stroma, respectively (Fig. 4e; Supplementary Fig. S4h), as CAF-FAP is enriched in FR^‒^ tumor core (*P* = 8.7E‒03) while CAF-C7 is specifically clustered at FR (*P* = 8.2E‒03). Besides, we noticed a slighter enrichment of HSC in the tumor core of FR^+^ tumors (Fig. 4e, *P* = 0.052). However, CAF-MYH11 and pericytes showed no significant difference in FR and TS_FR^‒^ area between FR^+^ and FR^‒^ samples, thus may not strongly correlate with stroma archetypes (Supplementary Fig. S4i, j).

Interestingly, CAF-FAP, CAF-C7, and HSC exhibited functional similarities to published perturbation-related fibroblast subtypes: inflammatory CAFs (iCAF), myofibroblast CAF (myCAF), and antigen-presenting CAF (apCAF)^57^, respectively (Fig. 4f; Supplementary Fig. S4k). CAF-FAP showed higher expression of ECM fibrillar collagen *COL11A1* (Fig. 4b), which agreed with the dense and twisted fibers observed in the tumor stroma of FR^‒^ HCCs (Fig. 2i). CAF-C7 highly expressed ECM basement membrane genes (such as collagen IVs and laminins) (Fig. 4b), which was in line with the sheet-like fiber alignment at FR (Fig. 2i). Furthermore, 25 differentially expressed genes (DEGs) of CAF-C7 were also found in FR-specific proteins and CAF-FAP exhibited 23 upregulated genes shared with TS_FR^‒^-specific proteins (Supplementary Fig. S4l). We also found HSC mainly showed upregulated pathways related to metabolic terms, including retinoid metabolism (Supplementary Fig. S4m), consistent with the metabolic preference in the TS_FR^+^ (Fig. 2f). The consistent findings across different spatial modalities collectively suggest a potential association between CAF subsets and stromal architecture.

Furthermore, we identified spatial DEGs of CAF-FAP and CAF-C7 along the tumor-margin-normal axis at four defined spatial zones (Fig. 4g; Supplementary Table S4). Gene set variation analysis (GSVA) revealed increased ECM organization in CAF-C7 in the FR zone and upregulated inflammation in CAF-FAP at the tumor core compared with other zones (Supplementary Fig. S4n). These results suggested fibroblast functional plasticity in different spatial contexts. Notably, CAF-FAP and CAF-C7 both showed upregulated chemokine signaling pathway (Fig. 4h), but showed high expression of pro-inflammatory genes (*CCL2*, *CXCL12*, *GDF15*, and *LIFR*) at tumor core and pro-fibrotic genes (*TNFRSF12A*, *TGFBR1*, *TGFBR2*) at FR, respectively. Also, spatial DEGs of CAF-FAP and CAF-C7 overlapped with ECM proteins enriched in TS_FR^‒^ (S100A11, LSGAL9, ISG20) and FR (FBLN5, DCN, COL4A1) (Figs. 2e, 4i).

We next investigated the relationship of CAF-FAP and CAF-C7 with patient prognosis. Using RNA-seq data from the TCGA dataset, we observed that CAF-FAP correlated with significantly worse patient outcomes in multiple cancer types, including HCC (Fig. 4j; Supplementary Fig. S4o and Table S4). In line with the TCGA results, recent scRNA-seq studies also suggested that FAP-expressing fibroblasts exhibited tumor-promoting roles in lung cancer, colorectal cancer, and PDAC^47,58,59^. Next, we examined the human HCC TMAs and confirmed the minimally overlapped expression of FAP (representing CAF-FAP) and PDGFRA (representing CAF-C7) on fibroblasts (VIM^+^) at the protein level (Fig. 4k; Supplementary Table S4). We found patients with higher CAF-FAP (FAP^+^VIM^+^) at tumor core or lower CAF-C7 (PDGFRA^+^VIM^+^) at tumor margin exhibited worse survival (tumor core, *n* = 154, *P* = 0.036; tumor margin, *n* = 148, *P* = 0.037; Fig. 4l). Collectively, we reasoned that CAF-FAP and CAF-C7 are associated with two stromal archetypes implicated in HCC, respectively, and their spatial balance may have a strong clinical implication.

### RUNX1 and USF2 govern distinct stromal programs in CAF-FAP and CAF-C7

**Fig. 5:**
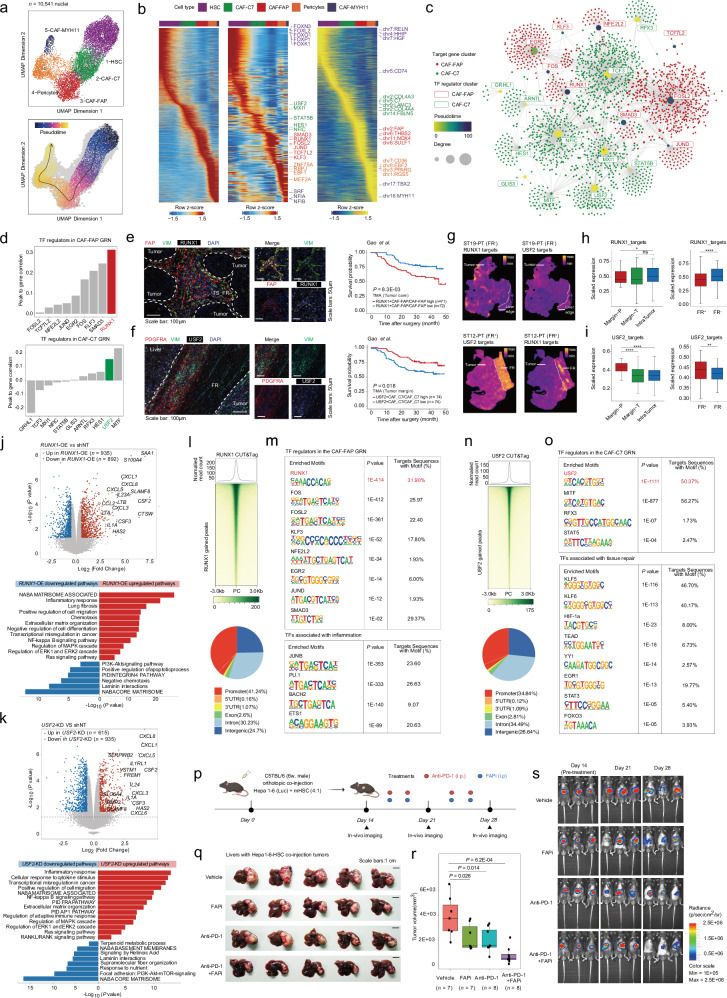
Gene regulatory networks of distinct CAF subpopulations in HCC. **a** UMAP representations and pseudotime trajectories of fibroblast subsets by integrated snRNA-seq and snATAC-seq. **b** Changes in highly variable peaks (left), transcription factor (TF) motif binding activities (middle), and gene expression score (right) along the pseudotime trajectories of fibroblast subsets. Marker TFs and marker genes were shown and colored by cluster. **c** GRN of CAF-FAP and CAF-C7. Each node represents a TF (regulator) or a gene (target). TFs were colored according to the pseudotime value and target genes were colored by their corresponding fibroblast subcluster. **d** Bar plot showing the mean peak-to-target-gene correlation (FDR < 1 × 10^3^, VarQATAC > 0.25, VarQRNA > 0.25) of TF regulators in the CAF-FAP GRN (upper) and CAF-C7 GRN (lower). **e** Representative images of multi-plex imaging for CAF-FAP subset and identified TF RUNX1 and survival analysis based on immunostaining scores of RUNX1^+^CAF-FAP/CAF-FAP in cohort 2 (Tumor core, *n* = 143). Log-rank test. Scale bars: 100 μm and 50 μm. **f** Representative images of multi-plex imaging for CAF-C7 subset and identified TF USF2 and survival analysis based on immunostaining scores of USF2^+^CAF-C7/CAF-C7 in cohort 2 (Tumor margin, *n* = 148). Log-rank test. Scale bars: 100 μm and 50 μm. **g** Spatial visualization of RUNX1 and USF2 target gene expression density in representative FR^+^ and FR^‒^ samples. **h** Comparison of target gene expression density of RUNX1 among different spatial zones of representative samples and between FR^+^ and FR^‒^ groups (FR^+^, *n* = 18; FR^‒^, *n* = 10). Wilcoxon test (ns: *P* > 0.05; *P < 0.05; **P < 0.01; ***P < 1E‒03; ****P < 1E‒04). **i** Comparison of target gene expression density of USF2 among different spatial zones of representative samples and between FR^+^ and FR^‒^ groups (FR^+^, *n* = 18; FR^‒^, *n* = 10). Wilcoxon test (ns: *P* > 0.05; *P < 0.05; **P < 0.01; ***P < 1E‒03; ****P < 1E‒04). **j** Volcano plots (upper) and enrichment (lower) of DEGs in human hepatic satellite cells (LX-2) with or without *RUNX1* overexpression (*RUNX1*-OE) at RNA level. Scale bars: 5 mm. **k** Volcano plots (upper) and enrichment (lower) of DEGs in human hepatic satellite cells (LX-2) with or without *USF2* knockdown (*USF2*-KD) at RNA level. **l** Heatmap of the RUNX1 CUT&Tag-seq profiles of LX-2 cells (upper), pie plot showing genomic distribution of binding sites (lower). **m** Tables illustrating the RUNX1 CUT&Tag motif enrichment analysis. **n** Heatmap of the USF2 CUT&Tag-seq profiles of LX-2 cells (upper), pie plot showing genomic distribution of binding sites (lower). **o** Tables illustrating the USF2 CUT&Tag motif enrichment analysis. **p** Schematic diagram of orthotopic Hepa1-6-mHSC co-injection model and drug interventions. **q** Representative images of harvested livers from orthotopic HCC models receiving different treatments. Scale bars: 1 cm. **r** Boxplots showing the tumor volume by different regimens (vehicle, *n* = 7; FAPi, *n* = 7; Anti-PD-1, *n* = 8; Anti-PD-1+FAPi, *n* = 8). Wilcoxon test. **s** Representative images of in-vivo imaging from orthotopic HCC models receiving different treatments.

Although we observed spatially and functionally heterogeneous phenotypes between CAF-FAP and CAF-C7, the molecular mechanisms regulating their specific gene expression remain largely unknown. Recent efforts probed that fibroblast phenotypic shifts in cancer contexts may be accompanied by epigenetic changes^30,60^. Thus, we integrated matched snRNA-seq and snATAC-seq data to study the gene regulatory programs driving fibroblast spatial heterogeneities in HCC. After quality control and filtration, 10,541 fibroblasts remained (Supplementary Fig. S5a, b) and were clustered into five distinct subsets (Fig. 5a, b; Supplementary Fig. S5c‒e and Table S5) that recapitulated the five cell states identified by snRNA-seq (Fig. 4a). Pseudo-time ordering predicted a single-lineage trajectory (Fig. 5a), which is consistent with the subcluster enrichment along the tumor-margin-liver axis (Fig. 4e), suggesting that intratumor CAF-FAP and marginal CAF-C7 may arise from HSC at the distal liver, consistent with previous reports in pre-clinical models in HCC^53,61^.

Next, we inferred a fibroblast gene regulatory network (GRN) to gain insights into the mechanisms regulating CAF-FAP and CAF-C7 polarization. Clustering of GRN resulted in 2 modules corresponding to the potential transcriptional factors (TFs) regulating CAF-FAP and CAF-C7 (Fig. 5c; Supplementary Fig. S5f and Table S5). To identify TFs with potential positive regulatory roles, we performed peak-to-gene correlation analysis and found RUNX1 emerged as the top-ranking TF in the CAF-FAP GRN, while USF2 ranked as one of the top candidate TFs in the CAF-C7 GRN (Fig. 5d). Given MITF’s established role as a regulator in melanocytes and melanoma, particularly its repression of genes associated with the extracellular matrix and focal adhesion pathways^62^, we opted to prioritize investigation of USF2 in the CAF-C7 module, alongside RUNX1 in the CAF-FAP module.

Multi-plex IHC confirmed co-localization of RUNX1 with FAP protein at the tumor core and USF2 with PDGFRA expression at the tumor margin (Fig. 5e, f; Supplementary Table S5). Moreover, increased RUNX1 expression on intratumor CAF-FAP was correlated with poor prognosis, and increased USF2 expression on marginal CAF-C7 was correlated with better prognosis (Fig. 5e, f). The prognostic significance of both RUNX1 and USF2 aligns with the subclusters they are enriched in (Fig. 4j‒l). Thus, we assumed them as potential regulators of CAF-FAP and CAF-C7. The importance of RUNX1 has been documented in CAFs isolated from human CRC^63^ and CAFs derived from mouse breast cancer models^64^, while previous studies have not reported the regulatory role of USF2 in fibroblasts in human cancer. Nevertheless, previous studies have demonstrated a tumor suppressor role of USF2 in inhibiting tumor proliferation in vitro^65^ and reducing tumorigenicity in vivo^66^, which resonates with the tumor-restraining properties observed for CAF-C7.

Consistent with the tumor-promoting role of CAF-FAP, functional enrichment analysis of RUNX1-targeted genes pointed to those involved in increased fibroblast proliferation, cell migration, and inflammation (Supplementary Fig. S5f). Contrarily, USF2-targeted genes were associated with stress response and cell survival (Supplementary Fig. S5f), which may prevent malignant transformation and ECM resolution caused by inflammatory stimuli, such as stress or aging, to restrain tumor growth. Visualization of the RUNX1 and USF2 target genes on Stereo-seq revealed that these genes are exclusively expressed in stromal at tumor core and tumor margin (Fig. 5g‒i), in line with spatially exclusive localization of CAF-FAP and CAF-C7 (Fig. 4c, d).

To examine the functional roles of RUNX1 and USF2 in vitro, we performed bulk RNA-seq on perturbated human liver fibroblasts (LX-2) with *RUNX1* overexpression (*RUNX1*-OE) or *USF2* knockdown (*USF2*-KD). Unsurprisingly, we observed a higher CAF-FAP signature in *RUNX1*-OE cells and a lower CAF-C7 signature in *USF2*-KD cells (Supplementary Fig. S5g). DGE analysis (|Log_2_FC | > 0.5, *P* < 0.05) showed that *RUNX1*-OE significantly promoted the expressions of 935 genes that are closely related to inflammation and ECM remodeling, including *CXCL1*, *CXCL8*, *IL1A*, *HAS2*, which were also upregulated after *USF2*-KD. Functional annotation indicated that both *RUNX1*-OE and *USF2*-KD increased the expressions of genes involved in inflammatory response, chemotaxis, cell migration, and MAPK pathway activation while decreasing the expressions of genes involved in the laminin interactions, apoptotic process, PI3K-AKT pathway (focal adhesion) and retinoic acid metabolism (Fig. 5j, k; Supplementary Fig. S5h and Table S5), consistent with the transcriptional profiles of CAF-FAP and CAF-C7 (Fig. 4f). Next, we studied fibroblast proliferation and migration to test the behavior differences caused by TF alternations (Supplementary Fig. S5i‒k). Combined with RNA-seq analysis, we hypothesized that USF2 may inhibit fibroblast growth by suppressing TGFβ signaling and activating retinoic acid metabolism while RUNX1 may promote fibroblast migration through proteolysis.

To investigate the genomic distribution of RUNX1 and USF2, we conducted cleavage under targets and tagmentation followed by sequencing (CUT&Tag-seq) in LX-2 cells (Fig. 5l‒o). Both RUNX1 and USF2 regulatory sites were predominantly enriched in promoter regions (41.24% and 34.84% respectively) (Fig. 5l, n). Interestingly, we found that 8 out of 9 TFs in the CAF-FAP GRN were significantly enriched in RUNX1 binding sites (Fig. 5m), while 4 out of 11 TFs in the CAF-C7 GRN were significantly enriched in USF2 binding sites (Fig. 5o). This suggests the possibility of co-binding of RUNX1 and USF2 on these sites to further regulate CAF functions. Furthermore, RUNX1 binding motifs were mainly associated with inflammatory gene categories, including JUNB, BACH2, and ETS1 (Fig. 5m), indicating RUNX1’s role as a pro-inflammatory transcriptional activator. Similarly, USF2 was found to bind to TFs associated with tissue repair, such as KLF5, HIF-1a, YY1, and FOXO3 (Fig. 5o), supporting the potential roles of USF2 in mediating wound healing-like processes.

Guided by the evidence that CAF-FAP may promote local inflammation and tumor progression, we reasoned inhibiting CAF-FAP may help attenuate HCC development in vivo. Using two independent C57BL/6 orthotopic HCC xenograft assays, we demonstrated that inhibiting FAP by Ac-Gly-BoroPro limited tumor growth, and this synergized with anti-PD-1 treatment (Fig. 5p‒s; Supplementary Fig. S5l‒n). H&E and Masson staining on the tumor margin of both orthotopic models confirmed the absence of FR, supporting an FR^‒^ phenotype (Supplementary Fig. S5o). To preliminarily explore the mechanism underlying the effect of FAP inhibition on CAF-FAP, we conducted multiplex imaging and revealed a significant reduction in the frequency of CAF-FAP among total CAFs following treatment (Supplementary Fig. S5p, q). This suggests that FAP inhibition may mitigate HCC progression by reducing the abundance of CAF-FAP. Previous in vivo studies conducted in lung and colon cancer^67^ also demonstrated the efficacy of FAP inhibition in reducing tumor stroma and suppressing overactivated fibroblasts.

Gathered, we found that RUNX1 and USF2 may be crucial TFs regulating the opposing functions of CAF-FAP and CAF-C7. Targeting CAF-FAP may be a promising new strategy for controlling HCC and sensitizing the ICB treatment.

### Multicellular immune hubs surrounding and occupying the HCC tumor mass

**Fig. 6:**
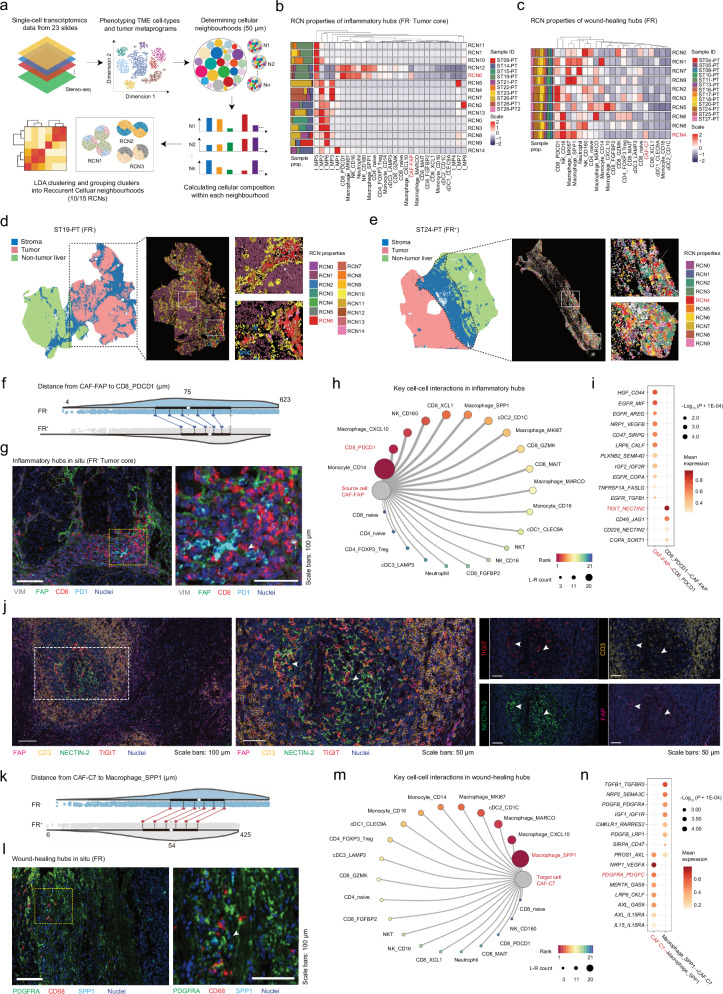
Coordinated CAF-immune interactions in HCC stroma. **a** Schematic of the RCN analysis. Annotated single-cell data from TME cell types and tumor metaprograms (t_MPs) were added to 23 Stereo-seq slides. The latent Dirichlet allocation (LDA) model was trained with a 50 μm proximity radius and subsequently grouped by K-means clustering into 10 or 15 RCNs based on cellular composition and the frequency of occurrence. **b**, **c** RCN properties of inflammatory hubs at the tumor core of FR^‒^ tumors (**b**) and wound-healing hubs at the FR of FR^+^ tumors (**c**). Heatmap shows the abundance of cell types with each RCN and bar plots to the left of the heatmap represent the distribution of patients with each cluster. **d**, **e** Spatial visualization with regional magnification of the tumor core of FR^‒^ tumors (**d**) and the FR of FR^+^ tumors (**e**). Cells were colored based on their RCN class. Arrows mark the examples of inflammatory hubs (**d**) and wound-healing hubs (**e**). **f** Shift plots showing the distance between CAF-FAP and CD8_PDCD1 in the tumor core (*n* = 23; FR^+^, *n* = 13; FR^‒^, *n* = 10). Significance is calculated for percentiles of 30, 40, 50, 60, 70 by the Robust Harrell-Davis quantile estimator. The blue line represents a significant difference between FR^+^ and FR^‒^ tumors (*P* < 0.05, lower in FR^‒^ tumors), and the grey line represents non-significance for the percentile. **g** Representative images highlighting the co-localization of CAF-FAP (FAP^+^VIM^+^) and CD8_PDCD1 (CD8^+^PDCD1^+^) at tumor-occupying stroma. Arrows mark the examples of co-localization pairs. Scale bars: 100 μm. **h** Circus plot showing CD8_PDCD1 ranked second by counts of ligand‒receptor pairs among all presumable target cells of CAF-FAP in the tumor core. **i** Dot plot showing significant ligand‒receptor interactions between CAF-FAP and CD8_PDCD1 in the tumor core. The point size represents the log-normalized *P* value, while the filled color indicates the mean expression of the ligand‒receptor pairs. **j** Representative images showing the intercellular crosstalk between CD8_PDCD1 and CAF-FAP through the ligand‒receptor of NECTIN2‒TIGIT. Scale bars: 100 μm or 50 μm. **k** Shift plots showing the distance between CAF-C7 and Macrophage_SPP1 in the liver edge (*n* = 23, FR^+^, *n* = 13; FR^‒^, *n* = 10). Significance is calculated for percentiles of 30, 40, 50, 60, 70 by Robust Harrell-Davis quantile estimator. The red line represents a significant difference between FR^+^ and FR^‒^ tumors (*P* < 0.05, lower in FR^+^ tumors), and the grey line represents non-significance for the percentile. **l** Representative images highlighting the co-localization of CAF-C7 (PDGFRA^+^) and Macrophage_SPP1 (CD68^+^SPP1^+^) at tumor-surrounding stroma. Arrows mark the examples of co-localization pairs. Scale bars: 100 μm. **m** Circus plot showing Macrophage_SPP1 ranked first by counts of secreted ligand‒receptor pairs among all presumable target cells of CAF-C7 at the tumor margin. **n** Dot plot showing the ligand‒receptor pairs significantly upregulated between CAF-C7 and Macrophage_SPP1 at the tumor margin. The point size represents the log-normalized *P* value, while the filled color indicates the mean expression of the ligand‒receptor pairs.

As observed in the spatial multi-omics and bulk sequencing data, several pathways related to immune regulation and inflammatory signaling were upregulated in CAF-FAP and FR^‒^ tumors (Figs. 1j, k, 2f, 4f). We postulated that the opposing roles of CAF-FAP and CAF-C7 on tumor progression may also be associated with their interactions with the local immune cells. To determine how CAF-FAP and CAF-C7 interact with local TME, we performed recurrent cellular neighborhood (RCN) analysis^68^ on Stereo-seq to identify crucial cellular neighborhoods of CAF-FAP and CAF-C7 (Fig. 6a). To comprehensively include cell types in the RCN analysis, we further annotated tumor cells, T cells, and myeloid cells (Supplementary Fig. S6a‒d; Supplementary Table S6). Notably, the heterogeneity of HCC tumor cells was not fully characterized by previous single-cell studies^24,27,29^. Here, we identified 9 recurrent tumor metaprograms (t_MPs) in HCC and found that they were unevenly distributed between FR^‒^ and FR^+^ tumors (Supplementary Fig. S6e, f). Interestingly, t_MP5 (featured stress response) enrichment in FR^‒^ tumors was consistent across Stereo-seq, spatial proteomics, and bulk RNA-seq data (Supplementary Fig. S6g). Further hierarchical clustering of patients in cohort 2 using the signature genes of t_MPs resulted in three groups (Sub1-3) with different overall survival (Supplementary Fig. S6h, i). The t_MP5 was enriched in Sub2 with the worst prognosis, consistent with the poor survival observed in t_MP5-enriched FR^‒^ tumors (Fig. 1d, e). These results suggest the potential role of stress response in shaping stromal architecture and clinical outcomes.

Bulk analysis revealed the upregulation of KRAS, glycolysis, and hypoxia in FR^‒^ HCCs (Fig. 1j), indicating stem-cell-like characteristics. Consistently, we observed a significant elevation in embryonic stem cell-like signatures^69^ (including the OCT4, SOX2, and NANOG targets) and the canonical HCC stem-cell marker *PROM1*^70^ in FR^‒^ HCCs (Supplementary Fig. S6j-m). Examining *PROM1* expression among different t_MPs, we found it most enriched in t_MP7 (De-differentiation) (Supplementary Fig. S6n) that is also enriched in FR^‒^ tumors (Supplementary Fig. S6f). Aligned with our observation of T cell exclusion in the tumor core of FR^‒^ tumors (Figs. 1k, 3h), we found an exclusive enrichment of *DDR1*, a collagen I receptor, within FR^‒^ tumors (Supplementary Fig. S6o, p), suggesting a potential interplay between tumor and stroma in FR^‒^ HCCs, particularly with CAF-FAP characterized by high expression of *COL1A1* (Fig. 4b). The interaction of COL1A1 and DDR1 has been implicated in promoting immune exclusion in breast cancer^71^. Further exploration of tumor‒stroma interactions may elucidate the underlying mechanisms of HCC tumor cell heterogeneity and shed light on the factors driving aggressiveness and immune evasion.

Subsequent RCN analysis revealed that CAF-FAP was enriched explicitly in RCN6 at the FR^‒^ tumor core, while CAF-C7 was mainly enriched in RCN4 at the FR^+^ tumor margin (Fig. 6b, c; Supplementary Fig. S6q). RCN6 was mainly localized in tumor stroma, where CAF-FAP was spatially proximal to t_MP5, CD8_PDCD1, neutrophil, Monocyte_CD14, and Macrophage_CXCL10 (Fig. 6b, d), which featured by inflammation and immune exhaustion. Similarly, RCN4 was enriched in marginal stromal-rich areas, where CAF-C7 was accompanied by Macrophage_SPP1 (Fig. 6c, e). Macrophage_SPP1 has been previously reported to play a crucial role in tissue remodeling by actively interacting with fibroblasts^72^, implying that Macrophage_SPP1 may foster local ECM remodeling together with CAF-C7. Interestingly, RCN4 tended to cluster as small patches, sporadically distributed, and intermingled with other RCNs (Fig. 6e), which shared a similar pattern with fibrotic niches in cirrhotic livers^51^. According to the collective function of cell types in RCN6 and RCN4, we termed them “inflammatory hubs” and “wound-healing hubs,” respectively.

Further spatial proximity quantification analysis, multi-plex imaging (Supplementary Table S6), and ligand‒receptor analysis suggested that CD8_PDCD1 and CAF-FAP were spatially co-localized and functionally correlated (Fig. 6f‒h). Specifically, CD8_PDCD1 emerged as the predominant subpopulation within inflammatory hubs (Supplementary Fig. S7a) and was positioned closer to CAF-FAP compared to Monocyte_CD14 in the intratumoral zone of FR^‒^ tumors (Supplementary Fig. S7b). Within inflammatory hubs CD8_PDCD1 preferentially expressed TIGIT and CAF-FAP expressed NECTIN2 as a cell surface ligand of TIGIT (Fig. 6i, j; Supplementary Fig. S7c). The interaction of TIGIT with NECTIN2 has been documented in human cancer single-cell studies, including HCC^73^ and neuroblastoma^74^. NECTIN2 encodes a membrane ligand that interacts with TIGIT, thereby conferring inhibitory signals. Notably, blockade of TIGIT has demonstrated efficacy in preventing T cell exhaustion and boosting antitumor immunity^75^. However, the specific expression of NECTIN2 on certain subsets of CAFs in HCC, and its potential impact on CD8^+^ T cell antitumor immunity, remain incompletely understood.

To investigate whether NECTIN2 expression in fibroblasts mediates CD8^+^ T cell dysfunction through interaction with TIGIT, we performed NECTIN2 overexpression in LX-2 cells (Supplementary Fig. S7d) and conducted co-culture experiments with CD8^+^ T cells isolated from human PBMCs. Our findings reveal that NECTIN2 overexpression led to a decrease in the secretion of GZMB in CD8^+^ T cells (Supplementary Fig. S7e). Furthermore, we observed that inhibitory antibodies blocking the interaction between TIGIT and NECTIN2 (Ociperlimab and Tiragolumab) largely reversed the immune suppression of CD8^+^ T cells (Supplementary Fig. S7e). To further investigate the interaction dynamics, we conducted high-content cell imaging in co-culture experiments (Supplementary Fig. S7f). Our results demonstrate that TIGIT^+^CD8^+^ T cells exhibited a significantly stronger aggregation tendency around NECTIN2^+^ fibroblasts over time, as measured by Ripley’s K function (Supplementary Fig. S7g). These results provide further evidence for stromal‒immune interaction mediated by the NECTIN2-TIGIT axis.

In wound-healing hubs, we confirmed that Macrophage_SPP1 and CAF-C7 exhibited significant proximity (Fig. 6k,l; Supplementary Fig. S7h) and inter-cellular communication (Fig. 6m). CAF-C7 highly expressed *PDGFRA*, while Macrophage_SPP1 had elevated expression of *PDGFC* (Fig. 6n). PDGFC may directly trigger PDGFRA signaling in CAF-C7, in keeping with our data showing increased deposition of ECM and activation of PI3K signaling at tumor margin (Fig. 4h; Supplementary Fig. S5f).

Together, these results suggest that the opposing function of CAF-FAP and CAF-C7 may be partly due to their polarization of the local TME into different functional stromal hubs. Future investigation into selectively targeting inflammatory hubs while preserving or enhancing wound-healing hubs should rejuvenate anti-tumor immunity in HCC.

### Stromal inflammatory hubs across different cancer types

**Fig. 7:**
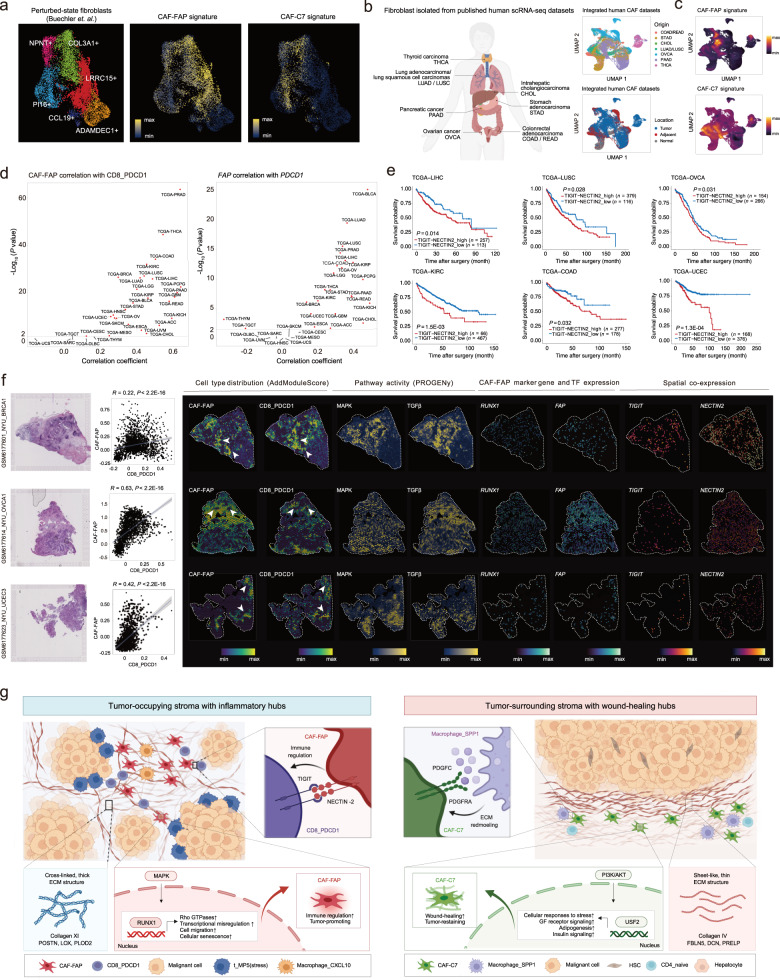
Validation of stromal inflammatory hubs Across various cancer types. **a** UMAPs showing tissue origin of fibroblasts from human perturbed-state fibroblasts atlas (left) and their expression of CAF-FAP (middle) and CAF-C7 (right) signature. **b** Schematic diagram of integrating scRNA-seq data of human fibroblasts collected from different cancer types and locations. **c** Estimated CAF-FAP (upper) and CAF-C7 (lower) abundance in pan-cancer fibroblasts, showing the expression of subcluster signature among cancer types. **d** Pearson correlation between CAF-FAP and CD8_PDCD1 abundance and between *FAP* and *PDCD1* gene expression of TCGA data across 32 solid tumor types. Cell abundance was quantified by mean expression of the top 50 DEGs. Pearson correlation. **e** Kaplan-Meier plot showing the overall survival across 6 cancer types from TCGA based on the mean expression of *TIGIT* and *NECTIN2*. Log-rank test. **f** Characterization of the inflammatory hubs in published ST data of human breast, ovarian, and endometrial cancer, showing paired histology, spatial distribution, and correlation of CAF-FAP and CD8_PDCD1, pathway activity, marker gene expression, and ligand‒receptor pairs. Pearson correlation. **g** Schematic diagram of the two major stromal archetypes (FR^+^ and FR^‒^) in HCC, displaying broad differences between FR^+^ and FR^‒^ tumors regarding fibroblast heterogeneity, multicellular interactome, and ECM properties.

Although we described the role of CAF-FAP and CAF-C7 with distinct stromal hubs, whether they exist under different stromal-perturbated settings remains to be elucidated. Using recent single-cell data consisting of human perturbation-specific activated fibroblast states^76^, we found that CAF-FAP partly overlapped with LRRC15^+^ CAFs from PDAC and NSCLC patients, indicating a cross-cancer prevalence. Contrarily, CAF-C7 exhibited less similarity to CAFs but was more closely related to pro-fibrotic NPNT^+^ alveolar fibroblasts and PI16^+^ universal fibroblasts (Fig. 7a). Next, we obtained public single-cell data on fibroblasts from tumor, normal, and adjacent non-cancer tissues across 7 common malignancies^77–83^ to examine the role of CAF-FAP and CAF-C7 across different cancer types (Fig. 7b; Supplementary Table S7). Fibroblasts with high CAF-FAP scores were clustered together and mainly accumulated in tumors, in contrast, cells with high CAF-C7 scores were enriched in adjacent areas of most cancer types (Fig. 7c; Supplementary Fig. S8a), which is consistent with tissue distribution of CAF-FAP and CAF-C7 in HCC (Fig. 4c, e).

As we noted before, multiple immunosuppressive pathways and molecules were upregulated in the dense stroma of FR^‒^ tumors compared with FR^+^ tumors (Fig. 2f). In the RNA-seq data of the TCGA, a higher CAF-FAP score is positively correlated with the increased CD8_PDCD1 score across 27 cancer types (*P* < 0.05; Fig. 7d; Supplementary Fig. S8b and Table S7), aligning with the physical juxtaposition of CAF-FAP and CD8_PDCD1 in HCC tumor stroma (Fig. 6f, g) and indicating a universal distribution of inflammatory hubs across multiple cancer types. In line with the pan-cancer results, we found in HDTVi HCC models (MYC_sgP53) with higher Fib_Fap frequency exhibited greater Cd8_Pdcd1 abundance (Supplementary Fig. S8c‒e) and an impaired response to immunotherapy (Supplementary Fig. S8f), suggesting MYC_sgP53 may be a counterpart of inflammatory hubs in human tumor stroma and serve as a modeling system for further mechanistic study. Also, we found co-expression of *FAP* and *PDCD1* in 22 different cancer types (*P* < 0.05; Fig. 7d; Supplementary Fig. S8g and Table S7). Thus, co-targeting FAP and immune checkpoints may be an effective combination therapy design, such as combining FAP-directed chimeric antigen receptor T cell with ICB^84^.

Analysis of the TCGA RNA-seq data further showed that *TIGIT* and *NECTIN2* high expression was correlated with more progressive disease across 14 cancer types, including HCC (Fig. 7e; Supplementary Table S7), suggesting targeting TIGIT‒NECTIN2 to inhibit stromal‒immune interactions may be a potent alternative to directly reduce CAF-FAP to fuel ICB response. Later, utilizing published ST data (10X Visium), we spatially mapped cell type abundance and biological processes within the inflammatory hubs to three human cancers (i.e., breast cancer, ovarian cancer, and endometrial cancer) and ICB-treated HCCs^6,85,86^. In the pan-cancer dataset^85^, we found increased TGF-β and MAPK signaling in the tumor stroma was spatially associated with higher CAF-FAP and CD8_PDCD1 abundance. Moreover, *RUNX1* (CAF-FAP-specific TF) and *TIGIT*-*NECTIN2* expression were also upregulated in CAF-FAP and CD8_PDCD1 colonization areas (Fig. 7f). Subsequent validation on 2 independent HCC ST cohorts confirmed stroma distribution of inflammatory hubs in the patients with poor ICB response^6,86^ (Supplementary Fig. S8h‒k). Moreover, in five bulk RNA-seq cohorts that were administered ICB in other cancer types^87^, higher CAF-FAP signature is also correlated with unfavorable overall survival (Supplementary Fig. S8l).

Collectively, these results confirmed that the spatial and functional dependency between CAF-FAP and CD8_PDCD1 were broadly present in tumor stroma, extending our observations and clinical implications in human and mouse HCC to diverse origins of major cancer types.

## Discussion

Tissue architecture is critical for tumor initiation, progression, and treatment response^88^. Our study obtained a multimodal spatial atlas of human HCC, identifying two distinct stromal archetypes that significantly impact clinical outcomes. Our findings highlight that CAFs are spatially heterogeneous in human HCC, emphasizing their roles in shaping stromal architecture and organizing functional hubs (Fig. 7g).

Stromal architecture has been relatively overlooked in the clinical management and spatial research of HCC. In previous AI-powered image analysis^35^, we systematically analyzed tumor cells, necrosis, or immune cells. However, compared with these components, stroma architecture is more feasible to identify in both manual inspection and AI recognition. By connecting histological stromal patterns with clinical metrics on well-documented HCC datasets, we defined FR as a positive prognostic indicator, distinguishing two stromal archetypes (FR^‒^ and FR^+^). Comparing the multi-omics profiles of the two archetypes, we hypothesized that FRs may promote anti-tumor immunity by reducing inflammation, as marked by decreased stromal activation, T cell exclusion, and myeloid inflammation. Additionally, neither tumor mutation nor liver fibrosis prominently impacts the existence of FR, suggesting the biological differences of stromal archetypes may root in CAF heterogeneity^33^. Together, the integrated molecular data provide mechanistic clues for observed stromal heterogeneity and guide the subsequent design of high-throughput spatial profiling. Interestingly, we also found higher Wnt/β-catenin signaling in FR^+^ HCCs and higher IFN signaling activation in FR^‒^ HCCs, which warrant further mechanistic investigation. Although prior studies have suggested a correlation between Wnt/β-catenin signaling and immune exclusion, as well as a lack of response to immunotherapy^89^, recent studies have shown that some patients with Wnt activation can exhibit enrichment of activated CD8^+^ T cells^39^, and CTNNB1-mutant ICB responders may display enhanced anti-tumor immunity^90^, highlighting the complex role of Wnt activation in the immune landscape and treatment response. While IFN signaling is involved in T cell activation and effector functions, prolonged IFN signaling may also lead to immune suppression in chronic inflammatory conditions, including cancer^91–93^. Moreover, IFN signaling plays a role in ICB resistance^92–95^ and inhibiting IFN signaling in cancer can enhance therapeutic efficacy^91,92,94,96^. Despite that the overall immune dysfunction may lead to decreased IFN levels in tumors, persistent inflammation increased in FR^‒^ HCCs may drive transcriptomics or epigenetic changes in tumor cells to maintain chronic IFN stimulation, promoting disease progression or resistance programs.

HCC-associated stromal architectures are heterogeneous in terms of the spatial ECM textures. In addition to ECM abundance, the ECM alignment could mediate T cell migration and motility^32^. We demonstrated that dense and cross-linked structures in the tumor-occupying stroma of HCC were associated with immune exclusion, probably by reducing matrix porosity or chemoattract permeability. In contrast, loose and sheet-like structures in the tumor-surrounding stroma are prone to be lymphoid-permissive by providing tracks for immune migration and permitting T cell entry into the tumor nests. Although several ECM-based prognostic features have been proposed across multiple cancer types, the exact proteins that sculpt different ECM structures remain largely unknown^45^. Our study presented a spatial proteomic ECM map and found extensive alterations of ECM proteins associated with stromal heterogeneity, which may inform the future development of therapeutic agents in HCC. Collectively, our proteomics data shed light on the complex stromal biology regarding spatial ECM composition and inspire future reconfiguration strategies.

Our spatial multimodal analysis discovered that CAF-FAP and CAF-C7 may account for the spatial, functional, and prognostic differences between two stromal archetypes, and we further validated their generalizability for various cancer types. Previous studies in lung cancer^47,97^ and pancreatic cancer^98,99^ also proved that targeting FAP-expressing CAFs can enhance anti-tumor immunity. Recently developed FAP-targeting bi-specific antibody (colorectal and pancreatic cancer)^100^, antibody-drug conjugate (ADC) (pancreatic, ovarian, and lung cancer)^101^, potently increased T cell Infiltration and control tumor growth in pre-clinical models. These CAF-FAP-targeted regimens may be further tested to explore their potential in synergizing HCC immunotherapy. Altogether, these results imply that restoring the CAF spatial balance has profound clinical implications.

Previous studies mainly focused on CAF-tumor cell interactions, suggesting that CAF metabolic reprogramming could promote tumor cells by providing nutrients^102–104^. However, targeting CAF-specific metabolic vulnerabilities was hampered by our current knowledge of the complex metabolic network in TME. In this study, we pivoted to the stromal‒immune interactions. We defined CAF-FAP-oriented inflammatory hubs and CAF-C7-dominated wound-healing hubs and confirmed their universal role in tumor biology. In inflammatory hubs, CAF-FAP may interact with CD8_PDCD1 and contribute to T cell suppression, as reflected by reduced production of GZMB. Moreover, CAF-FAP may also directly dampen cellular responses to TNF-α and IFN-γ, thereby shielding tumors cells from cytokine-induced hypoxic necrosis^97^. As the SHG imaging of TS_FR^‒^ and ECM profiles of CAF-FAP suggest, these fibroblasts may further impede CD8^+^ T cell function through knitting dense ECM fibers in tumor stroma to drive immune exclusion. Surprisingly, inflammatory hubs were also coupled with regional tumor programs, consistent with recent reports^23,33^, suggesting stroma‒immune interaction may be part of a more extensive communication network that exacerbates local inflammation. In wound-healing hubs, CAF-C7 may form a two-cell circuit with Macrophage_SPP1, creating a fibrotic barrier that limits tumor invasion and restrains tumor‒normal inflammation crosstalk. As “a wound that never heals”, tumor mass persistently invades liver tissue during progression, maintaining chronic inflammation intertwined with the scarring process that may finally lead to FR formation at the tumor‒normal interface. Interestingly, similar wound-healing hubs containing activated macrophages and ECM-remodeling fibroblasts were reported in human granulomas^105^. The ring surrounding granulomatous lesions can promote antibacterial activity while limiting tissue destruction, displaying a similar anti-inflammatory role as FRs in HCC. Therefore, we propose that defined wound-healing hubs may share development programs with granulomas that warrant further investigation. Future development of rational experimental systems may uncover stimuli or mechanisms underlying stromal hub formation and maintenance, such as metabolic segregation^106^ or mechanical stress^88^.

In summary, we demonstrated multimodal profiling as a powerful tool to understand CAF heterogeneity and stromal‒immune interactions, informing stroma-targeted strategies that may benefit clinical practice in HCC and beyond. However, our study has certain limitations that should be considered. The LX-2 cell line may not fully capture the biological heterogeneity of CAFs within the HCC tumor microenvironment compared to primary CAF cultures. Further functional studies were required to elucidate the mechanisms by which RUNX1 and USF2 impact fibroblast subpopulations and tumor biology. How different stromal architectures arose and evolved during tumor progression or upon treatments remains elusive. Improvements in modeling systems may reveal the principals governing stromal compartments across temporal axes. Time-resolved tracking may elucidate how CAF-FAP and CAF-C7 dynamically evolve during HCC progression, distinguishing key CAF subpopulations from bystanders and revealing the dynamics of stromal organizations. Furthermore, as stroma architecture was interconnected throughout the serial sections of the invasive front, studying stromal heterogeneity in a 3D manner may bring additional biological insights.

## Materials and methods

### Cohort description

Fresh tumor, adjacent non-tumor, and resected tumor‒normal interface specimens and FFPE tissue blocks were obtained from 58 patients (fresh sample, *n* = 28; FFPE sample, *n* = 30) who underwent primary curative resection at Zhongshan Hospital (Shanghai, China). All samples included in our study were diagnosed by two experienced pathologists, confirming HCC. Mixed HCC-ICC were rigorously excluded from our study. A summary of the study cohort including clinical and pathological information, along with the sequencing experiments conducted, is provided in Supplementary Tables S2, S3. Two experienced pathologists evaluated key histopathological features for patient classification. This study was performed in accordance with the Ethics Committee of the Zhongshan Hospital (B2021-065R), and informed consent was obtained from each enrolled patient. The study complies with all relevant ethical regulations.

### Cell lines

The LX-2 cell line was purchased from Pricella Biotechnology Co. Ltd. The Hepa1-6 cell line was obtained from Dr. Daming Gao (Shanghai Institute of Biochemistry and Cell Biology, Chinese Academy of Sciences). Both the LX-2 and Hepa1-6 cells were cultured in DMEM media (Gibco) supplemented with 10% FBS (Gibco), 100 U/mL penicillin, and 100 μg/mL streptomycin (Gibco) at 37 °C under 5% CO_2_, and tested routinely for mycoplasma contamination.

### Animal models

Four-to-six-week-old male C57BL/6 mice were obtained from Shanghai Model Organisms Center, Inc. for orthotropic implantation models. The experiments were approved by the Institutional Animal Care and Use Committee (IACUC) of Shanghai Model Organisms Center, Inc. (2023-0014-06). The construction of HDTVi models was performed under the approval of the Institutional Animal Care and Use Committee (IACUC) of the Shanghai Branch of Beijing Vital River Laboratory Animal Technologies Co. Ltd. (2017-0014). All mice were maintained in a specific pathogen-free (SPF) facility and received humane care according to the Guide for the Care and Use of Laboratory Animals criteria of the National Academy of Sciences.

### Artificial intelligence-powered spatial analysis

We develop an immune-stroma identification model powered by a weakly supervised deep-learning framework to analyze the spatial distribution of heterogeneous immune cells and stroma components. Whole-slide images (WSIs) were digitalized with a 20× objective lens and a sliding step size of 128, divided into 256 × 256 pixels-sized grids using Openslide (https://github.com/openslide/openslide-python).

We randomly selected 50 WSIs from cohort 2. Areas of these WSIs were delineated by pathologists and classified by spatial location (normal, stroma, tumor) using a prior-proposed classification model^35^ for further analysis. The representative intra-tumoral area was marked by immune cell distribution into 3 classes (high/moderate/low), corresponding to TLS-rich, immune-infiltrating, and immune-cold areas, while for stroma and normal, typical areas were marked into 2 classes (high/low). The annotated WSIs were randomly divided into a training set (80%) and a test set (20%). According to the annotations, masks were generated and divided into various patches.

We used the Van-small algorithm with an attention mechanism to train the classification mode. We also applied color augmentation and sample-balanced sampling to improve training efficiency. The parameters of our model were initialized using the ImageNet dataset before training and the AdamW optimizer (https://pytorch.org/docs/stable/generated/torch.optim.AdamW.html) was applied in the process. To be specific, the immune score was defined as: ${\rho }_{1}=\,\frac{I{S}_{1}+I{S}_{2}}{{S}_{1}+{S}_{2}}$, and the stroma score was defined as ${F}_{1}=\,\frac{{S}_{1}}{{S}_{1}+{S}_{2}}$. ${S}_{1}$: area of stroma, ${S}_{2}$: area of parenchymal cells, $I{S}_{1}$: area of stromal infiltration, $I{S}_{2}$: area of parenchymal infiltration.

### Subclassification of boundary area into spatial zones

Referred to our previous findings^38^, we defined tumor margin into 4 zones, including “tumor core” (> 500 μm from the tumor border inside the tumor area), “distal” (> 500 μm from the tumor border inside the non-tumor area), “tumor edge” (≤ 500 μm from the tumor border in the tumor area) and “liver edge” (≤ 500 μm from tumor border inside the non-tumor area). For FR^+^ tumors, the “liver edge” was specialized to the entire width of the FRs, and the “distal” covers the area beyond the FR accordingly.

### Preprocessing of multi-omics data from CPTAC

All patients in cohort 2 were enrolled in the Clinical Proteomic Tumor Analysis Consortium (CPTAC) project. The multi-omics expression data for cohort 2^34^ were acquired from the National Omics Data Encyclopedia (NODE) (https://www.biosino.org/node) with the accession code OEP000321. The Log (FPKM)-normalized values were employed for subsequent analytical procedures.

### Calculating gene signature scores

The stromal score was calculated from RNA-seq data using the ESTIMATE algorithm (version 2.0.0). Gene signature scores were used to assess the differential enrichment of functional pathways. We used the ssgsea() function of the GSVA R package (version 1.46.0) for evaluation. Gene sets were obtained from the MSigDB database (version 7.0). The significance threshold was set to a *P* value < 0.05.

### Laser capture microdissection

For spatial proteomic analysis, 30 FFPE blocks were selected for LCM using PALM Microbeam (Zeiss). Briefly, FFPE-embedded tissue was sectioned into 10 μm-thick sections and mounted on PEN 1.0 membrane slides (Zeiss). One adjacent section/case of each dissected sample was stained with H&E to aid in the identification of stroma, tumor, and liver tissue. LCM experiment was performed within 12 h after the sections were prepared on PEN slides. Approximately 5000–10,000 cells/sample were cut and collected in Tris-HCl solution (Sigma-Aldrich) for protein extraction for each tissue type. All samples were heated at 95 °C for 5 min, spun down, and then stored at −80 °C for further processing.

### Proteomic sample preparation

For proteomics sample preparation, each sample was suspended in 100 μL lysis buffer containing 50% (v/v) Acetonitrile (ACN), 300 mM Tris-HCl (pH 8.0), lysed by sonication (Qsonica) for 16 cycles for 80% intensity and then centrifuged at 12,000× *g* for 2 min at room temperature (RT). Next, each sample was heated at 90 °C for 90 min without shaking and then centrifuged at 12,000× *g* for 2 min at RT. The samples were reduced with 10 mM tris(2-carboxyethyl)phosphine, alkylated with 40 mM 2-chloroacetamide, and incubated for 30 min at 56 °C with 1500 rpm. Subsequently, the samples were evaporated by the Speedvac centrifuge (Thermo Fisher Scientific) for about 40 min to a volume of 20 μL. The proteins were diluted with 100 μL digestion buffer containing 10% (v/v) ACN in water and digested at 37 °C and 1200 rpm overnight after adding 1 μg of Trypsin/Lys-C Mix (Promega). For proteome measurements, the digested peptides were acidified with 20 μL 10% (v/v) Trifluoroacetic acid (TFA) and desalted using SDB-RPS (3 M) StageTips. In brief, peptides were loaded directly onto the StageTips, washed sequentially with 1% (v/v) TFA in isopropanol and 0.2% TFA, and finally eluted with 60 μL 80% acetonitrile, 5% ammonia. The resulting peptides were dried in a SpeedVac centrifuge and reconstituted in 0.1% formic acid. Peptide concentrations were measured optically at 205 nm.

### LC-MS/MS analysis

The nanoElute (Bruker) coupled with a timsTOF Pro mass spectrometer (Bruker) via nano-electrospray ion source (Bruker) was used to measure all samples. 200 ng peptides were loaded on a 20 cm × 75 μm homemade column (Dr. Maisch). The column was heated to 50 °C using an in-house-manufactured oven. The gradient time is 60 min, and the gradient was set as 2%–4% B in 1 min, 4%–26% B in 47 min, 26%–32% B in 5 min, 32%–90% B in 2 min at a flow rate of 250 nL/min, followed by 90% B maintained for 5 min at a flow rate of 300 nL/min. Source capillary voltage was set to 1500 V in positive ion mode and dry gas flow to 3 L/min at 180 °C. For data-independent acquisition, samples were acquired with a diaPASEF method consisting of 14 cycles, including a total of 28 mass-width windows (25 Da width, from 452 to 1152 Da) with 4 mobility windows each, making a total of 56 windows covering the ion mobility range (1/K0) from 0.76 to 1.29 Vs/cm^2^. The acquisition time of each PASEF scan was set to 100 ms with a near 100% duty cycle, which led to a total cycle time of around 1.59 s.

### Proteomic data analysis

DIA-NN (version 1.8.1) was used to process diaPASEF data with a library-free mode search against the *Homo sapiens* SWISS-PROT reference proteomes database (release 2021_09, 20588 entries, https://www.uniprot.org/). The false discovery rate (FDR) was set to 1% at the precursor and protein levels. N-terminal methionine excision was enabled. Cysteine carbamidomethylation was set as a fixed modification and methionine oxidation as a variable modification. The rest of the parameters were kept as default.

Missing quantitative values were imputed using the K-nearest neighbor (KNN) algorithm in the DreamAI R package (version 0.1.0, https://github.com/WangLab-MSSM/DreamAI). Proteins detected in less than half of the samples of stromal (LS, TS, and FR) and non-stromal components (liver and tumor) were removed from downstream analysis. Partial Least Squares Discriminant Analysis (PLS-DA) was performed by using the R package mixOmics (version 6.22.0). The results were visualized in 3D with confidence ellipses using rgl (version 1.1.3, https://github.com/dmurdoch/rgl).

The R package Limma (version 3.54.2) was applied to identify differentially expressed proteins. The significance threshold was set to a FDR < 0.05. Functional and pathway enrichment analyses were performed using Metascape (version 3.5) or ClusterProfiler (version 4.6.2). The networks of enriched pathways were constructed by Cytoscape (version 3.7.2).

### 3D reconstruction of stromal components

For 3D reconstruction of stromal components, 150 tissue slides of each sample were consecutively sectioned (4 μm in thickness), stained by H&E, and scanned at a magnification of 40× (~0.5 μm per pixel) to generate serial images. Pyvips (version 2.2.2, https://pypi.org/project/pyvips/) was used to save a downsized copy of each image, corresponding to 0.5 μm per pixel, using nearest neighbor interpolation. For each sample, the center image was selected as the reference, and both rigid and elastic registration were applied to align other images within the sample to this reference. Then, we performed registration on down-sampled versions of high-resolution histological slices. A pair of preprocessed tissue images were matched and registered using feature detection. Rigid registration was performed sequentially, aligning each image strictly with the reference image. After rigid registration, the tissue mask bounding box extracted high-resolution tissue images from the tissue slide and performed elastic registration on them. Elastic registration involves finding 2D displacement fields X and Y, which distort the “moving” image to align with the “fixed” image by optimizing a metric. As the displacement fields are non-uniform, they can distort the image to align local features better. Once the transformation parameters were found, the original high-resolution tissue slices were transformed using libvips (version 1.0.9, https://github.com/libvips/libvips) to obtain the registered images.

Slices of the registered tissue images were generated at a scale of 20× to produce 512 × 512 × 3 image patches. Using the training weights, stroma across tissue patches in the sample was identified by a prediction model described in our prior study^35^. The predicted labels of the patches were stitched together at low resolution to form the labeled tissue image of the entire image. The multi-label images created by deep learning were merged into a 3D point cloud matrix using the H&E image registration results. The labeled image is read using OpenCV (version 4.3.0, https://github.com/opencv/opencv-python), and the read-in labeled images were merged to form a 3D point cloud using numpy, with different tissues having different RGB color schemes and saved in the xyzrgb format.

### SHG microscopy

SHG images were captured from unstained, uncovered, and hydrated whole slides of HCC tissues. We used two-photon imaging (Bruker) containing a 5-PMT array to characterize collagen content and structure. HCC tissue was imaged using a 20× water immersion lens for SHG and autofluorescence. Following a 1 h laser warm-up period, a femtosecond multiphoton laser was used to emit an excitation wavelength of 1045 nm onto the tissue and then separated with a filter set at 550 nm. Images were acquired and generated at a resolution of 2048 × 2048 pixels. Data normalization, background filtering, and *Z*-stacking reconstruction of raw SHG images were performed using the Fiji software.

### DNA extraction and WES

Samples were taken and enzymatically disrupted to a size of 180‒250 bp. Then, DNA fragments were end-polished, A-tailed, and ligated with the full-length adapter for Illumina sequencing. Libraries were hybridized with the liquid phase with a biotin-labeled probe (Agilent), then magnetic beads were used with streptomycin to capture the exons of genes. Captured libraries were enriched in a PCR reaction, and the products were purified using AMPure XP beads (Beckman Coulter). The libraries were analyzed for size distribution using agarose gels and were quantified using real-time PCR. The clustering of the index-coded samples was performed on a cBot Cluster Generation System using the Novaseq 6000 S4 Reagent Kit (Illumina) according to the manufacturer’s instructions. After cluster generation, the DNA libraries were sequenced on the NovaSeq 6000 platform (Illumina), and 150 bp paired-end reads were generated.

### WES data processing and tumor purity estimation

The paired-end reads were initially mapped to the UCSC human reference genome (hg19) and underwent cross-contamination analysis. Somatic SNVs and INDELs were also identified at least two kinds of software, including MuSE (version 1.0), Strelka2 (version 2.9.9), MutTect2, SomaticSniper (version 1.0.5.0), Lancet (version 1.0.7) and SvABA (version 0.2.1). Moreover, germline variants were removed by using multiple databases, including the 1000 Genomes, the genomAD databases (https://gnomad.broadinstitute.org/), the ExAC database (http://exac.broadinstitute.org), and the ClinVar database (https://www.ncbi.nlm.nih.gov/clinvar/). To evaluate tumor purity, we used PyLOH (version 1.4.3) at the genomic level and ESTIMATE (version 2.0.0) at the transcriptomic level.

### scRNA-seq (10X Genomics)

Specimens were systematically gathered and transported at 4 °C in RPMI before undergoing further processing. Upon transfer to a Petri dish on ice, segments with adipose, and necrotic components were meticulously excised. After the removal of residual blood using 4 °C PBS, the tissue underwent mincing into small fragments (2‒4 mm^3^) in preparation for enzymatic dissociation. The dissected tissue was then introduced into digestion tubes (Miltenyi Biotec), each containing 5 mL of an enzymatic digestion mix (Miltenyi Biotec), with 5 mL of digestion mix for 1 g of tissue. The enzymatic digestion was carried out using the tissue dissociator (Miltenyi Biotec), resulting in a single-cell suspension. Subsequently, the digestion mix underwent filtration through a 70-μm cell strainer (Falcon), followed by washing with RPMI containing 1% fetal bovine serum. The resultant cell suspension was centrifuged at 500× *g* for 7 min in a pre-cooled centrifuge at 4 °C to form a cell pellet. This pellet was resuspended in loading buffer (PBS containing 0.04% BSA), filtered through a cell strainer snap cap (Corning) into a 1.5-mL Eppendorf tube, and then centrifuged at 500× *g* for 2 min at 4 °C. The resulting pellet was resuspended in cold loading buffer and quantified using trypan exclusion. The suspension was subsequently diluted to a concentration of 1000 cells/mL. Libraries were constructed according to the provided 10× protocol and subjected to sequencing on the Illumina platform.

### snRNA-seq and snATAC-seq

#### snRNA-seq

The OCT-embedded tissue was stored at −80 °C and thawed on the ice before experiment. It was then sectioned into small pieces (0.5 mm^3^) in an ice-cold sterile petri dish and homogenized. Afterward, the tissue mixture was filtered through a 70-μm cell strainer (Falcon) for second-round homogenization. Then it was filtered through a 30 μm cell strainer (Sysmex), centrifugated at 500× *g* for 5 min at 4 °C and washed with a blocking buffer (1× PBS, 1% BSA, and 0.2 U/μL RNase inhibitor). The nuclei were obtained by centrifugation and resuspension with buffer containing 1× PBS and 0.04% BSA, checked and counted by DAPI (Beyotime) staining.

The DNBelab C4 scRNA Preparation Kit (MGI) was utilized to prepare snRNA-seq libraries following the manufacturer’s instructions. The concentration of the libraries was determined using a Qubit ssDNA Assay Kit (Thermo Fisher Scientific). The libraries were sequenced by the DNBSEQ-T1 sequencer (MGI) using a 30-bp read length for read 1 and a 100-bp read length for read 2.

#### SnATAC-seq

To generate snATAC-seq libraries, we used the DNBelab C4 scATAC Library Preparation Kit (MGI). The nuclei suspension remaining after processing for the 3′ snRNA-seq assay was used for snATAC-seq library construction. We extracted nuclei with a Tn5 transposase coupling adapter. The transposed nuclei, along with barcoded beads, were encapsulated in droplets. Subsequent preamplification, bead collection to capture ATAC fragments, and secondary amplification were carried out according to the manufacturer’s protocol. The generated library was quantified by the Qubit ssDNA Assay Kit (Thermo Fisher Scientific). The libraries were then sequenced by the DNBSEQ-T1 sequencer (MGI) using a 70-bp read length for read 1 and a 50-bp read length for read 2.

### Stereo-seq

#### Tissue preparation

Fresh HCC tissue was collected in 30 min after surgical resection. We rinsed the tissue block using pre-cooled PBS solution at 4 °C, removed fat and necrotic areas, and quickly embedded it in OCT Compound (Sakura), followed by snap frozen. The tissue embedded in OCT was stored at a temperature of ‒80°C until it was ready for further processing. For each sample, 10 sections measuring 20 mm each were trimmed, collected, and lysed using TRIzol reagent (Invitrogen). The lysed sections were then used to determine the RNA integrity (RIN). RIN values were determined using a 2100 Bioanalyzer (Agilent). Samples with an RIN value > 7 were assessed as qualified and then used for further ST experiments.

#### Tissue sectioning, fixation, staining, and imaging

The Stereo-seq ST sequencing library was constructed using Stereo-seq capture chips (BGI Shenzhen) with spatial coverage of 1 cm × 1 cm area. The probes on the capture chip are 220 nm in diameter and have a center-to-center distance of 500 nm, providing up to 4 × 10^8^ capturing spots/chip. Serial cryosections of 10 um thickness were cut from the OCT-embedded tissue using a CM1950 cryostat (Leica). One piece of cryosection was placed on the capture chip, stained with the Qubit ssDNA Assay Kit (Thermo Fisher Scientific), and prepared for Stereo-seq sequencing, while 1 adjacent cryosection next to the captured section was stained using H&E for histological assessment. Both the H&E and ssDNA-FITC slides were scanned by a digital scanner (Motic).

#### Tissue permeabilization, cDNA synthesis, and sequencing

After image acquisition, the tissue section on the Stereo-seq chip underwent permeabilization using 0.1% pepsin (Sigma) in 0.01 N HCl buffer. The section was then incubated at 37 °C for 14 min and subjected to reverse transcription at 42° C. After in situ incubation overnight, the tissue section was gently removed by subjecting it to a tissue removal buffer at a temperature of 42 °C for 30 min. The chips containing reversely transcribed cDNA were then incubated with cDNA release enzyme treatment for 3 h at 55 °C and further amplified using KAPA HiFi Hotstart Ready Mix (Roche). A total of 20 ng of the amplified cDNA was then fragmented using Tn5 transposase at a temperature of 55 °C for 30 min. This was followed by a PCR reaction. The PCR products were then purified using Ampure XP Beads (Beckman Coulter) for DNB generation and sequenced with single-end 35 bp + 100 bp using an DNBSEQ-T1 sequencer (MGI). RNase-free SSC buffer (Thermo Fisher Scientific) was used as the washing buffer in the above-mentioned experiments.

### scRNA-seq data processing

For droplet-based sequencing data analysis, we utilized the Cell Ranger software (version 2.3, https://github.com/10XGenomics/cellranger) to align and quantify the data against either the GRCh38 human reference genome or the GRCm38 mouse reference genome. The resulting raw expression matrices were then processed using SoupX (version 1.6.1) to eliminate ambient RNA contamination with default parameters. The SoupX-corrected matrices were then processed using the Seurat v4 R package (version 4.2.0, https://satijalab.org/seurat/). Cells with fewer than 200 genes or a mitochondrial gene ratio over 25% were removed. Then, the Chord R package (version 2.0.1) was used to filter doublets. We normalized the data by the LogNormalize() function and identified the top 2000 highly variable genes (HVGs) for further analysis. We applied the Harmony R package (version 0.1.1) for batch removal and subclusters were annotated by unsupervised clustering.

### snRNA-seq data processing

The snRNA-seq data were first aligned to the human genome GRCh38 using the DNBelab C4 scRNA-seq pipeline (version v3.0, in-house). We further processed the data using similar strategies in scRNA-seq data analysis. Briefly, we removed ambient RNA contamination, filtered low-quality cells (< 200 detected genes and > 25% mitochondrial gene ratio), excluded doublets, normalized the data, identified top 2000 HVGs, and performed batch removal for downstream analysis. Functional clusters were identified and annotated based on the expression of known marker genes and gene enrichment analysis.

### Stereo-seq data quality control, filtering, and pre-processing

For Stereo-seq raw data, initial reads were decoded by the ST_BarcodeMap algorithm (version 0.0.1, https://github.com/BGIResearch/ST_BarcodeMap), and were filtered for adapter sequence and low-quality reads using fastp (version 0.23.4). The mapped reads were aligned to the reference genome GRCh38 via STAR (version 2.7.11a), and then annotated and processed using handleBam (version v1.0.0, https://github.com/BGIResearch/handleBam). The Exon transcripts were determined by counting reads that overlapped with the exon region by more than 50%. Intron transcripts were annotated for reads that had less than 50% overlap with the exon region but possessed overlapping sequences with the adjacent intron sequence. Any remaining reads that did not fall into these categories were classified as intergenic transcripts. The exonic and intronic reads were used to generate a CID-containing expression profile matrix. By employing the Lasso function provided on the BGI Stereomics platform (http://stereomap.cngb.org/), we extracted the raw spatial expression matrix data corresponding to the tissue region, guided by ssDNA and H&E staining. The raw matrix was then converted into the bin50-level matrix (50 × 50 spots, i.e., 25 × 25um). For quality control, the median number of gene types per bin50 for all chips should be over 500. The remaining spots with more than 25% mitochondrial counts were filtered by Seurat v4 (version 4.2.0) for downstream analysis.

### Stereo-seq data normalization and spatially confined clustering

We used the SCTransform() function from Seurat v4 (version 4.2.0) to normalize data from different samples. We performed unsupervised clustering by SpaGCN (version 1.2.5) and manually annotated each acquired cluster. Three recurrent subregions (tumor, non-tumor liver, stroma) were observed among different samples.

### Stereo-seq spot selection, image alignment, iterative cell segmentation, and benchmarking

Single-cell segmentation of Stereo-seq data was performed by our self-written algorithm, Iterative Stereo-seq. In our procedure, we first calculated the initial threshold for watershed segmentation, filtering the noise of nuclear DAPI staining images. Then, we used a modified watershed algorithm to separate nuclear from the paired image. Specifically, to segment the smudged regions, we designed another parameter to compare nuclear and around lightness for a better definition of the boundary of the nuclei. After we found the nuclear position, the coordinates of each nuclei position were used to extend in matched Stereo-seq RNA spot image until met the cell size threshold (2500 for HCC spots and 900 for non-HCC spots, annotated by performing unsupervised clustering at bin50 resolution), or no other spot was included. Also, original Stereo-seq RNA data were calculated by Log(tanh(count)) to avoid influence by outliers.

We compared our algorithm with Pci-seq and Iterative HMinima separately. We could not compare our method with others straightforwardly because the lack of annotated cancer Stereo-seq datasets at single-cell level. Instead, we used nuclei spot image data from the 2018 Data Science Bowl as shared input. All parameters of the 2 benchmarking methods were set to default parameters.

### TME cell type annotation on Stereo-seq

To quantify the major TME cell types within the stroma region, we used SPOTlight (version 0.1.6) with the cell type-specific topic profiles from scRNA-seq. The cell type with the maximum predicted score of each ST cell was annotated as the final cell type. Fibroblast and immune subtypes were further annotated by referring to subcluster signature genes.

### Boundary detection and layer enrichment evaluation

The tumor‒normal boundaries were decided based on pathological annotation using paired H&E staining. A self-written method with Java was used to divide area and layers. In our program, we detected the tumor‒normal boundary line and then used it to expand 1 layer with a bin50 width from the previously defined layers’ edge and do iterations. The iteration kept running until the ultimate layer reached the threshold we set.

To study the cell type enrichment in each layer, we adopted the following processed steps. Firstly, the number of each cell type of each layer was achieved by skipping windows of five layers. Then to eliminate the effect of the area difference of each skipping layer, the number of each cell type of each layer was normalized for the area using the following formula:

$${\rm{normalized\; cell\; number}}=({\rm{cell\; number}}({\rm{i}})/{\rm{s}}({\rm{i}}))/\mathop{\sum }\limits_{k=1}^{n}{\rm{cell\; number}}({\rm{k}})/{\rm{s}}({\rm{k}})$$

Cell number(i) represents the number of each cell type of the i-layer, s(i) represents the area of the i- layer, and n represents the total layer number.

We adopted a sliding window approach to evaluate the cell type enrichment by layer. For major TME cells, the density was calculated relative to the total cell number. When calculating the enrichment of fibroblast subsets, the number of each subpopulation was divided by the total fibroblast count in situ.

### Identification of shared meta-programs in malignant cells

To elucidate robust transcriptional programs within tumor cells, we employed a consensus Non-Negative Matrix Factorization (NMF) procedure using the R package NMF (version 0.23), with the number of factors set to 10 and conversion of all negative values to 0. Subsequently, a signature was defined for each factor using the top 100 genes according to NMF analysis. A total of 230 signatures from 23 samples were aggregated, and hierarchical clustering was performed using the 1-Jaccard index as the distance metric, resulting in recurrent expression programs across HCC tumor cells as 9 t_MPs. For each t_MP, an expression score was calculated according to the expression of signature genes. Each t_MP was redefined using the top 30 feature genes (ranked by their correlation with the expression score). The top 50 feature genes within each t_MP were employed for classifying patients in cohort 2 to MP_subs, followed by survival analysis.

### Spatial DEG identification and expression pattern analysis

We divided tumor‒normal interface slides into 4 areas (i.e., “tumor core”, “tumor edge”, “liver edge”, and “distal”). The four areas of a single sample were aggregated to form pseudo-bulk gene expression by each area and used for spatial expression pattern analysis. The pattern analysis was performed utilizing the R package Mfuzz (version 2.58.0) with the mean expression of genes in 4 areas.

### snATAC data processing

snATAC-seq data were aligned to the human genome GRCh38. We obtained the fragment files using an in-house DNBlab C4 snATAC-seq platform (version v3.0) with the default parameters. Afterward, we utilized the ArchR package (version 1.0) for downstream analysis. To filter low-quality cells, we employed the following criteria: (1) transcription start site enrichment > 4 and (2) the number of unique fragments > 1000. Doublet scores were calculated with *k* = 10, and doublets were further filtered (filterRatio = 1). We then performed dimensionality reduction using latent semantic analysis on the processed matrix, followed by clustering and cell type annotation. To integrate snATAC-seq and snRNA-seq data, we combined ArchR with Seurat v4 (version 4.2.0). Cells from the 2 datasets were aligned according to their gene score matrix and gene expression matrix, generating a gene integration matrix. Single-cell observations from snATAC-seq were then labeled and annotated using the integration matrix with default parameters. Trajectory analysis was performed using the addTrajectory() function.

### Calling peaks and enriched motifs and computing motif activity

We generated pseudo-bulk group coverages based on cluster identities with ArchR (version 1.0). To calculate motif enrichment, we utilized the Cis-BP database (version 2.0.0), with a cutoff of FDR ≤ 0.1 and Log_2_FC ≥ 0.5. We used chromVAR (version 3.18) to generate the motif activity for every single cell with default parameters.

### Gene-regulatory network inference for fibroblasts

We calculate the correlation between chromatin binding accessibility and gene expression (peak-to-gene correlation) using the addPeak2GeneLinks() function in ArchR (version 1.0). Genes with a self-correlation greater than 0.3 were selected as candidate transcription factors. We referred to the hTFtarget database to identify the downstream target genes of candidate TFs. Transcription factor‒target gene networks were constructed using Cytoscape (version 3.7.2).

### Recurrent cellular neighborhood (RCN) analysis

Each annotated cell in Stereo-seq was queried for its neighborhood cell frequency within a radius of 50 μm. The generated cell-type frequency matrix was used for LDA analysis using gensim (version 4.3.0, https://pypi.org/project/gensim/). The number of latent motifs was determined as *n* = 10 for the liver edge and *n* = 20 for the tumor core. K-means clustering was subsequently performed to identify RCN across samples (*k* = 10 for the liver edge and *k* = 15 for the tumor core, respectively).

### Spatial proximity volume scoring

The distance of two interested cell types was measured by calculating the Euclidean distance between the selected cell and its nearest neighborhood. The Harrell-Davis quantile estimator was applied to test the 30‒70 quantiles between 2 groups (FR^+^ vs FR^‒^) with bias-corrected confidence intervals.

### Cell‒cell interaction analysis

We applied CellphoneDB to infer cell‒cell interaction between fibroblast cell subsets and TME cells. The potential interaction strength between two cell subsets was characterized by both the counts of significantly enriched ligand‒receptor pairs and the mean expression of ligand‒receptor pairs. The enriched ligand‒receptor interactions between two cell subsets were calculated based on permutation tests. We defined ligand‒receptor pairs as significant when *P* < 0.1.

### Multi-plex imaging, immunohistochemistry, Masson trichrome, and Sirius red staining

For Multi-plex imaging, the deparaffinized tissue sections or TMAs were heated for 12 min in EDTA buffer (pH 9.0) and then incubated with 3% H_2_O_2_ for 20 min. Afterward, the tissue slides were blocked in 2.5% normal goat serum for 20 min at RT before incubation with primary antibodies overnight at 4 °C. On the other day, the stained tissue was washed with TBS-T (0.1%Tween-20 and 1× TBS solution) (Sangon, Biotech) and further incubated with a secondary antibody for 30 min at RT, followed by fluorescent dyes for 10 min at RT. Detailed information on each panel design was provided in Supplementary Tables S3–S6. Cells were counterstained with DAPI (Thermo Fisher Scientific). Images were acquired by TissueFAXS Plus (TissueGnostics) or Vectra Polaris (Akoya Biosciences) and further analyzed by commercialized software, including Phenochart (version 1.0.12), Inform (version 2.4.8) and StrataQuest (version 7.0).

For immunohistochemistry, sections were incubated with the primary antibody at 4 °C overnight, washed with TBS-T, and incubated with the IHC secondary antibody. DAB (3,3-diaminobenzidine tetrahydrochloride) was used as the chromogen, and nuclei were counterstained with hematoxylin. According to the manufacturer’s instructions, Masson’s trichrome and Sirius red staining was performed using ready-to-use kits (Solarbio). All stained slides were scanned in brightfield by a digital pathology slide scanner (KFBIO).

### Gene editing of LX-2 human fibroblast cell lines

*RUNX1*-OE, *RUNX1*-KD, and *USF2*-KD LX-2 cell lines were generated by transfection of lentivirus plvx-GFP-2A-*RUNX1*-IRES-Puro (5E+07 TU/mL), pLKO5-GFP-puro-*RUNX1*-sh (1E+08 TU/mL), pLKO5-GFP-puro-*USF2*-sh (1E+08 TU/mL), respectively. Cell transduction was performed by incubating the LX-2 cells with viral supernatants and 8 ug/mL polybrene (MCE) for 12 h. Transinfected cells were selected by 5 μg/mL puromycin (MCE) treatment for 7 days.

For *NECTIN2* overexpression, the full cDNA (1452 bp) was amplified by Platinum Taq DNA Polymerase High Fidelity (Invitrogen) and cloned into mammalian expression vector pcDNA3.1(+) (Invitrogen). LX-2 cells were transfected with 1 μg/mL of either pcDNA3.1(+)-*NECTIN2* or control pcDNA3.1(+) per well in 6-well plates.

### Fibroblast proliferation assay

LX-2 cells were seeded in triplicate into a 96-well plate (10,000 cells/well), cultured overnight, and stimulated for 24 or 48 h with 10 ng/mL TGF-β1 (MCE), IL-1α (PeproTech), IL-6 (PeproTech), retinoic acid (MCE) or left untreated. Afterward, a 10 μL cell counting kit (Vazyme) was added to each well and incubated with cells in 37 °C for 2 h. The plate was then placed in a plate reader (BioTek) to detect the absorbance at 450 nm.

### Fibroblast migration assay

*RUNX1*-KD, *USF2*-KD, and sh-NT (normal control) LX-2 cells were detached and dissociated by trypsin (Gibco), washed extensively in PBS, and resuspended in DMEM/F12 serum-free growth medium (Gibco). 20,000 LX-2 cells were placed into Transwell inserts (Corning) in triplicates into 24-well plates containing DMEM plus 20% FBS (Gibco). Cells were then cultured for 24 h to migrate through the Transwell membrane. After 24 h of incubation, the inserts were withdrawn from the plates. The remaining cells and culture media above the Transwell membranes were removed by a cotton tip. The inserts were then fixed for 10 min, stained in crystal violet for 20 min, washed in PBS 3 times, and left dry. Images were acquired by a brightfield microscope, and the number of fibroblasts was counted manually using a cell counter.

### Immunocytochemistry staining and confocal imaging

For immunofluorescence staining of fibroblasts cultured in vitro, cells were fixed in 4% paraformaldehyde (Sangon Biotech) for 15 min at room temperature, permeabilized in 0.1% PBS-Triton X-100 (Sangon Biotech) for 5 min on ice and then incubated with 1% BSA (Sangon Biotech) for 60 min at RT. The *RUNX1*-OE, *USF2*-KD, and control cells were then incubated with anti-human vimentin antibody (Abcam) overnight at 4 °C, followed by incubation with Alexa Fluor 594 secondary antibody (Abcam) at RT for 60 min. The *NECTIN2*-OE and control cells were first stained with rabbit anti-human NECTIN2 (Abcam) and mouse anti-human α-Tubulin (Proteintech) and then incubated with anti-mouse IgG Alexa Fluor 488 (Abcam) and anti-rabbit IgG Alexa Fluor 594 respectively (Abcam) at RT for 60 min. Finally, all stained cells were counterstained with DAPI (SouthernBiotech) before image acquisition. Confocal imaging was performed using an imaging system with 60× objectives (General Electric).

### RNA-seq, data processing, and data analysis

Total RNA of *RUNX1*-OE, *USF2*-KD, and sh-NT (control) LX-2 cells were extracted using the TRIzol reagent (Invitrogen). Libraries were constructed and sequenced by the HiSeq X-Ten system (Illumina). Each library was performed with three biological replicates. Raw sequencing reads containing rRNA was filtered by mapping the sequencing reads to the rRNA database using Bowtie2 (version 2.3.4.1). The paired-end reads were further filtered by SOAPnuke (version 2.1.0). Bowtie2 was also used to map RNA reads to UCSC human transcriptome GRCh38. The expression levels of all genes and isoforms were estimated by RSEM (version 1.3.1) with default parameters. Limma (version 3.54.2) was used to identify DEGs. The significance threshold was set to a FDR < 0.05. Pathway enrichment analyses were performed using Metascape (version 3.5).

### High throughput CUT&Tag, DNA sequencing, and data analysis

#### RUNX1 and USF2 CUT&Tag and DNA library preparation

A total of 100,000 cultured LX-2 cells were detached and dissociated with trypsin, resuspended in DMEM, and washed twice gently with wash buffer (20 mM HEPES, pH 7.5; 150 mM NaCl; 0.5 mM Spermidine; 1× Protease inhibitor cocktail). 10 μL Concanavalin A-coated magnetic beads (Bangs Laboratories) were added per sample and incubated at RT for 10 min. Then, the supernatant was removed, and the remaining cells were resuspended in Dig-wash buffer (20 mM HEPES, pH 7.5; 150 mM NaCl; 0.5 mM Spermidine; 1× Protease inhibitor cocktail; 0.05% Digitonin; 2 mM EDTA) and incubated with anti-human RUNX1 antibody (Abcam) or anti-human USF2 (Novus Biologicals) or IgG control (Millipore) at 1:50 dilution overnight at 4 °C. After that, the primary antibody and control were removed using a magnet stand. The secondary antibody (Millipore) was diluted at 1: 1:100 in Dig-wash buffer. Cells were then incubated with secondary antibody at RT for 60 min, washed using the magnet, and resuspended in Dig-wash buffer. The pA-Tn5 adapter complex was diluted at 1:100 in Dig-med buffer (0.01% Digitonin; 20 mM HEPES, pH 7.5; 300 mM NaCl; 0.5 mM Spermidine; 1× Protease inhibitor cocktail) and incubated with resuspended cells at RT for 60 min. Upon incubation, cells were washed 2–3 times for 5 min each time in Dig-med buffer and resuspended in tagmentation buffer (10 mM MgCl_2_ in Dig-med Buffer) and incubated at 37 °C for 60 min. DNA was purified using phenol-chloroform-isoamyl alcohol extraction and ethanol precipitation. 21 μL DNA was mixed with 2 μL of a universal i5 and uniquely barcoded i7 primer to amplify DNA libraries. A volume of 25 μL NEBNext HiFi 2× PCR Master mix (New England Biolabs) was added and mixed. The sample was placed in a thermocycler with a heated lid using the following cycling conditions: 72 °C for 5 min (gap filling); 98 °C for 30 s; 14 cycles of 98 °C for 10 s and 63 °C for 30 s; final extension at 72 °C for 1 min and hold at 8 °C. Clean-up of the libraries was performed using XP beads (Beckman Counter).

#### DNA sequencing

The size distribution of libraries was determined by automated electrophoresis (Agilent), and libraries were mixed to achieve equal representation as desired. DNA sequencing was performed by the Novaseq 6000 System (Illumina) using 150 bp paired-end reads following the manufacturer’s instructions.

#### Data analysis

To process raw data, reads containing adapter or ploy-N were removed to generate clean reads. Further filtration according to the calculated Q20, Q30 and GC content resulted in high-quality clean reads, which were then aligned to reference human genome GRCh38 using the BWA program (version 0.7.15). The MACS2 package (version 2.2.9.1, https://pypi.org/project/MACS2/) was used for peak-calling from the BAM files with q-value < 0.05. HOMER (version 4.11) was applied to the generated peak file and the genome fasta for motif analysis. Peaks were annotated using the annotatePeaks.pl function. For defining differentially accessible peaks, peak files of each sample were first merged using BEDTools (version 2.28.0). The counts of the reads over the bed were then determined for each sample using the multicov function from BEDTools. Finally, differentially accessible peaks were assessed using DESeq2 (version 1.42.0) (|Log_2_FC| > 1 and *P* value < 0.05).

### Orthotopic xenograft assays

To establish the Hepa1-6-mHSC co-injection model, the mouse Hepa 1-6 Luciferase reporter HCC cell line was co-injected with primary hepatic satellite cells at a ratio of 4:1. The ratio of HCC to HSC cells was determined based on the tumor purity (i.e., the estimated fraction of cancer cells in a tumor tissue) distribution in FR^‒^ HCC, with a median purity of 0.8. Primary hepatic satellite cells were isolated from C57BL/6 mouse liver through density gradient centrifugation. The efficacy of these models was assessed using in-vivo imaging (Perkin Elmer) to measure and equalize the baseline tumor volume before commencing drug treatment on Day 14. Subsequently, mice received intraperitoneal (i.p.) injections of 200 μg of anti-mouse PD-1 antibody (BioXCell) twice a week, with or without 10 μg of Ac-Gly-BoroPro (MCE). In vivo imaging was conducted weekly to monitor the dynamic changes in tumor volume.

For Hepa1-6 tumor block transplantation, Hepa1-6 cells were initially subcutaneously injected into the right flanks of 6-week-old male C57BL/6 mice at a density of 2 × 10^6^ cells per mouse. After tumor establishment on Day 14, mice were euthanized, and the tumors were excised. The obtained subcutaneous tumors were then sectioned into tissue blocks measuring 2 mm × 2 mm × 2 mm and implanted into the left lobe of the liver in C57BL/6 mice. Following implantation, mice received intraperitoneal (i.p.) injections of 200 μg of anti-mouse PD-1 antibody (BioXCell) every other day, with or without 10 μg of Ac-Gly-BoroPro (MCE) or vehicle daily.

Murine livers were harvested 2 weeks post-initial implantation for evaluation. Tumor volume was calculated using the modified ellipsoidal formula: V = ½ (Length × Width^2^).

### Co-culture of LX-2 cells and CD8^+^ T cells and flow cytometric analysis

*NECTIN2*-OE and control LX-2 cells were seeded in 24-well plates (50,000 cells per well). CD8^+^ T cells were enriched from PBMCs of healthy donors by positive selection using magnetic cell separation (MACS, Miltenyi Biotec) and co-cultured with LX-2 cells (500,000 T cells per well). The *NECTIN2*-OE group was treated with the TIGIT inhibitor Ociperlimab (10 μg/mL, MCE) or Tiragolumab (10 μg/mL, MCE). Cells were harvested for flow cytometric analysis after 48 h of co-culturing. Harvested cells were initially stained with Zombie Yellow viability dye (Biolegend) and then stained according to the manufacturer’s instructions for surface markers. For intracellular staining, cells were permeabilized using fixation and permeabilization buffer (eBioscience). Flow cytometry was conducted using an LSRFortessa instrument (BD Biosciences) and analyzed using FlowJo software (version 10).

### High-content live cell imaging

*NECTIN2*-overexpressing (*NECTIN2*-OE) and control LX-2 cells were cultured in confocal dishes and stained with DIO (MCE) 24 h post-transfection. Live TIGIT^+^CD8^+^ T cells were sorted from PBMCs obtained from healthy donors. Prior to addition to the LX-2 culture, the sorted T cells were incubated with CFSE for 15 min at 37 °C. The confocal dishes were subsequently stained with Hoechst (Yeason) and placed into a high-content imaging analysis system (SpinSR10, Olympus). Microscopic images of the cells were captured every 30 min over a duration of 8 h to monitor cellular interactions and dynamics.

The Ripley’s K function was calculated by the Kest() function in the spatstat R package.

### HDTVi models

We chose recurrent driver genes in liver cancer patients and designed 20 combinations of genetic alternations with tumorigenic potential for HDTVi. Designed vectors were delivered into the hepatocytes by injecting a solution of sterile saline (0.9%) containing 10 μg pT3-based vector, 10 μg of px330-based CRISPR/Cas9 vector, and 2.5 μg of SB13-Luc vector. The volume of solution for each mouse was calculated by 10% of their body weight. The vector-containing solution was injected through the tail vein using a 3-mL syringe. After injection, the mice were monitored for tumor burden, treated with 200 μg of anti-mouse PD-1 antibody (BioXCell) or control every 2 weeks, and finally sacrificed according to tumor burden evaluation. The response to anti-PD-1 treatment was determined by overall survival. The murine livers were harvested at end point, and HCC tumor tissue was isolated and collected for subsequent scRNA-seq. All HDTVi models utilized in our study were FR^‒^.

### Analysis of scRNA-seq data from human perturbed-state and pan-cancer fibroblasts

We analyzed published cross-tissue human fibroblast datasets using Seurat v4 (version 4.2.0) (the same pipeline as in the original data analysis unless otherwise indicated). We retrieved cancer-associated fibroblasts from 7 common cancer types^77–83^ other than HCC and performed batch removal using Harmony (version 0.1.1) for downstream analysis. The CAF-FAP and CAF-C7 signature score was calculated by the AddModuleScore() function from Seurat v4 (version 4.2.0) using the top 50 DEGs (ranked by Log_2_FC) of CAF-FAP or CAF-C7 referred to our snRNA-seq data.

### Analysis of public ST datasets (10X Visium)

To investigate the role of inflammatory hubs among different cancer types and upon anti-PD-1 treatment spatially, we analyzed 3 published 10X Visium ST datasets respectively^6,85,86^. Downstream analysis was performed using Seurat v4 (version 4.2.0). Cell type abundance of CAF-FAP and CD8_PDCD1 were displayed by spatial expression of the top 50 DEGs (ranked by Log_2_FC) of CAF-FAP or CD8_PDCD1 derived from our original data. Signaling pathway activities were estimated by the R package PROGENy (version 3.17) with default parameters.

### Validation of CAF signatures and ligand‒receptor pairs in TCGA datasets

Bulk RNA-seq data comprising 32 solid tumor types of the TCGA datasets were obtained from the GDC portal (https://portal.gdc.cancer.gov/). CAF-FAP, CAF-C7, and CD8_PDCD1 scores were calculated using the mean expression of their signature genes. Correlation between CAF-FAP and CD8_PDCD1 score, FAP, and PDCD1 expression were estimated using the Pearson correlation coefficient.

### Statistical analysis

Statistical analysis methods for WES, proteomics, single-cell sequencing, and ST data analyses were mainly described and referenced in the respective method details. Standard statistical tests were used to analyze the clinical data, including but not limited to Student’s *t*-test, Chi-square test, Fisher’s exact test, Kruskal-Wallis test, Log-rank test, and Cox proportional hazards regression model.

For categorical variables vs categorical variables, Fisher’s exact test was used; for categorical variables vs continuous variables, the Kruskal-Wallis test and grouped Wilcoxon test were applied; and for continuous variables versus continuous variables, the Pearson correlation or Spearman correlation was used. Kaplan–Meier plots (Log-rank test) were used to describe overall survival. Variables associated with overall survival were identified using univariate Cox proportional hazards regression models. Significant factors in univariate analysis were further subjected to a multivariate Cox regression analysis in a forward LR manner. All statistical tests were two-sided, and statistical significance was considered when *P* < 0.05 unless otherwise indicated. *P* values were adjusted using the Benjamini-Hochberg procedure to account for multiple tests. All the analyses of clinical data were performed in R (version 4.3.0).

## Supplementary information

Supplementary figures

Supplementary Table S1

Supplementary Table S2

Supplementary Table S3

Supplementary Table S4

Supplementary Table S5

Supplementary Table S6

Supplementary Table S7

## Data Availability

The data of spatial proteome, Stereo-seq, scRNA-seq, snRNA-seq, snATAC-seq, WES, RNA-seq, and epigenomic profiling data generated in this study were deposited in NODE and can be accessed through: https://www.biosino.org/node/project/detail/OEP004110. It is also available through the CNGB Sequence Archive (CNSA) of China National GeneBank DataBase (CNGBdb) with accession number CNP0004214. Processed data files were provided in Supplementary Tables.
