# Supplementary material for: Stromal architecture and fibroblast subpopulations with opposing effects on outcomes in hepatocellular carcinoma
Source: Cell Discov. 2025 Jan 28;11:1. doi: 10.1038/s41421-024-00747-z (PMC11772884; doi:10.1038/s41421-024-00747-z)
Supplement: Supplementary file 1 — Supplementary figures [file 41421_2024_747_MOESM1_ESM.pdf]

Supplementary Fig. S1

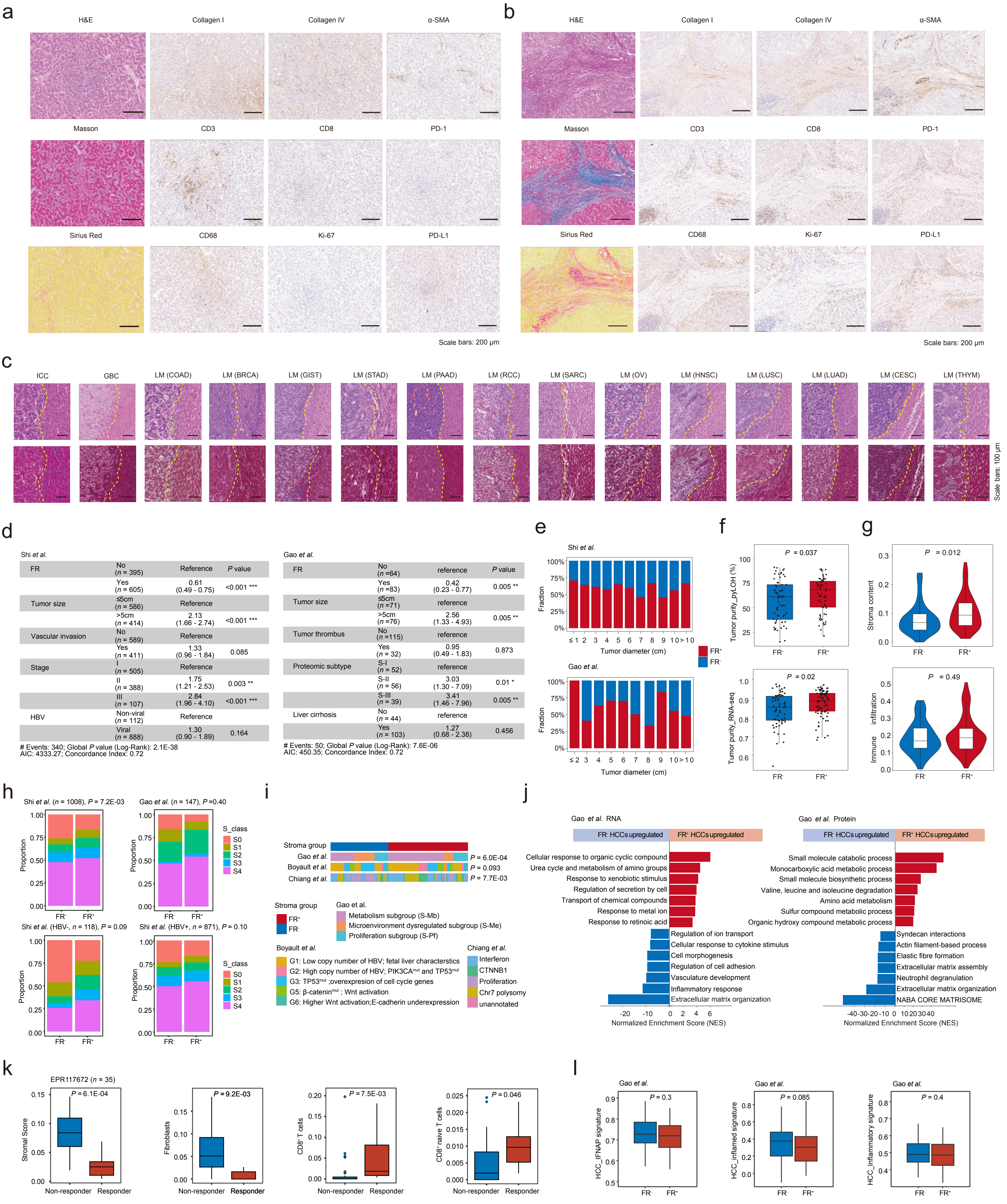

Shi *et al.*

FR	No (n = 395)	Reference	P value
	Yes (n = 605)	0.61 (0.49 - 0.75)	<0.001 ***
Tumor size	≤5cm (n = 586)	Reference	
	>5cm (n = 414)	2.13 (1.66 - 2.74)	<0.001 ***
Vascular invasion	No (n = 589)	Reference	
	Yes (n = 411)	1.33 (0.96 - 1.84)	0.085
Stage	I (n = 505)	Reference	
	II (n = 388)	1.75 (1.21 - 2.53)	0.003 **
	III (n = 107)	2.84 (1.96 - 4.10)	<0.001 ***
HBV	Non-viral (n = 112)	Reference	
	Viral (n = 888)	1.30 (0.90 - 1.89)	0.164

Events: 340; Global P value (Log-Rank): 2.1E-38
AIC: 4333.27; Concordance Index: 0.72

Gao *et al.*

FR	No (n =64)	reference	P value
	Yes (n =83)	0.42 (0.23 - 0.77)	0.005 **
Tumor size	≤5cm (n =71)	reference	
	>5cm (n =76)	2.56 (1.33 - 4.93)	0.005 **
Tumor thrombus	No (n =115)	reference	
	Yes (n = 32)	0.95 (0.49 - 1.83)	0.873
Proteomic subtype	S-I (n = 52)	reference	
	S-II (n = 56)	3.03 (1.30 - 7.09)	0.01 *
	S-III (n = 39)	3.41 (1.46 - 7.96)	0.005 **
Liver cirrhosis	No (n = 44)	reference	
	Yes (n = 103)	1.27 (0.68 - 2.38)	0.456

Events: 50; Global P value (Log-Rank): 7.6E-06
AIC: 450.35; Concordance Index: 0.72

Supplementary Fig. S2

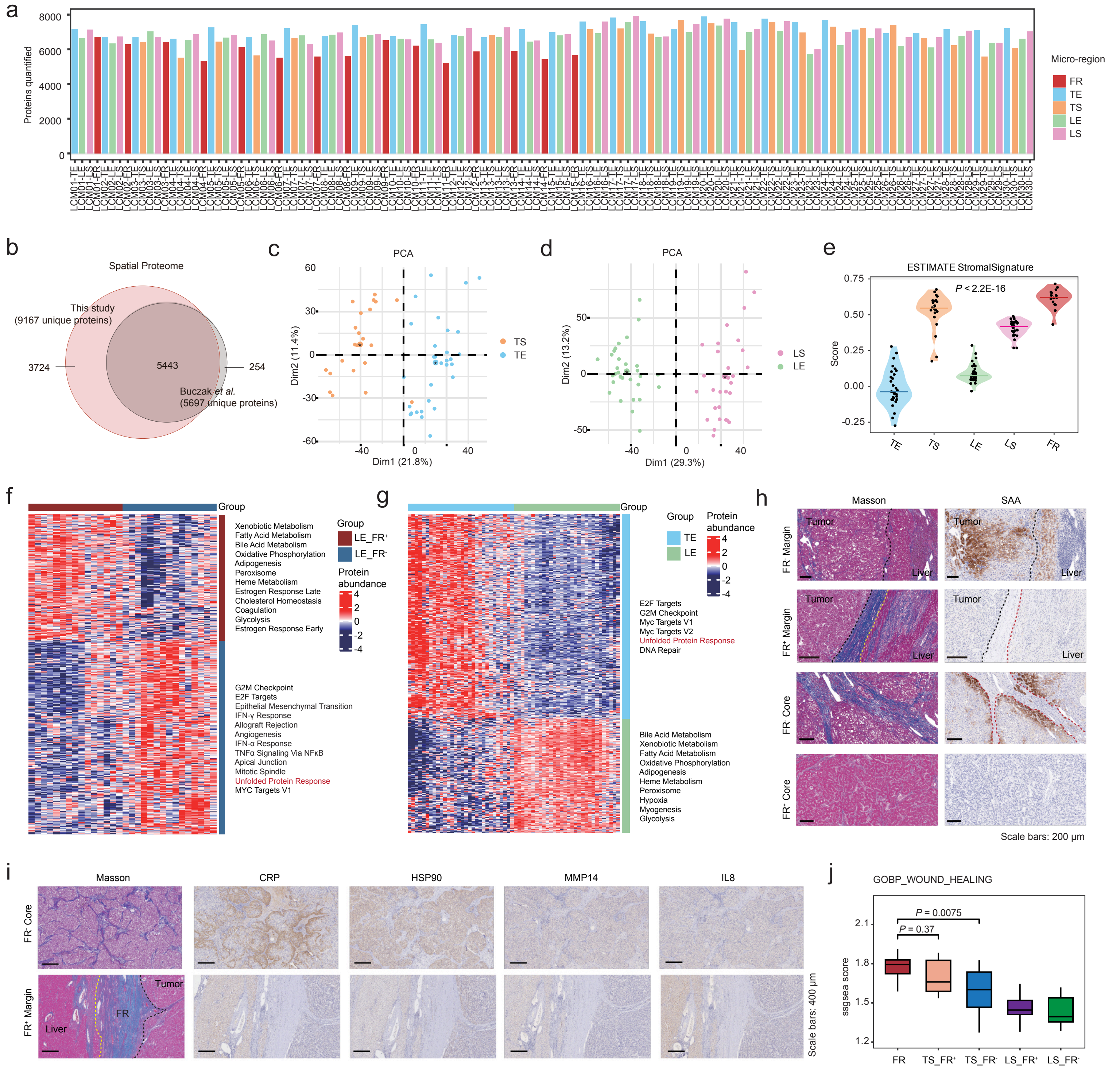

Supplementary Fig. S3

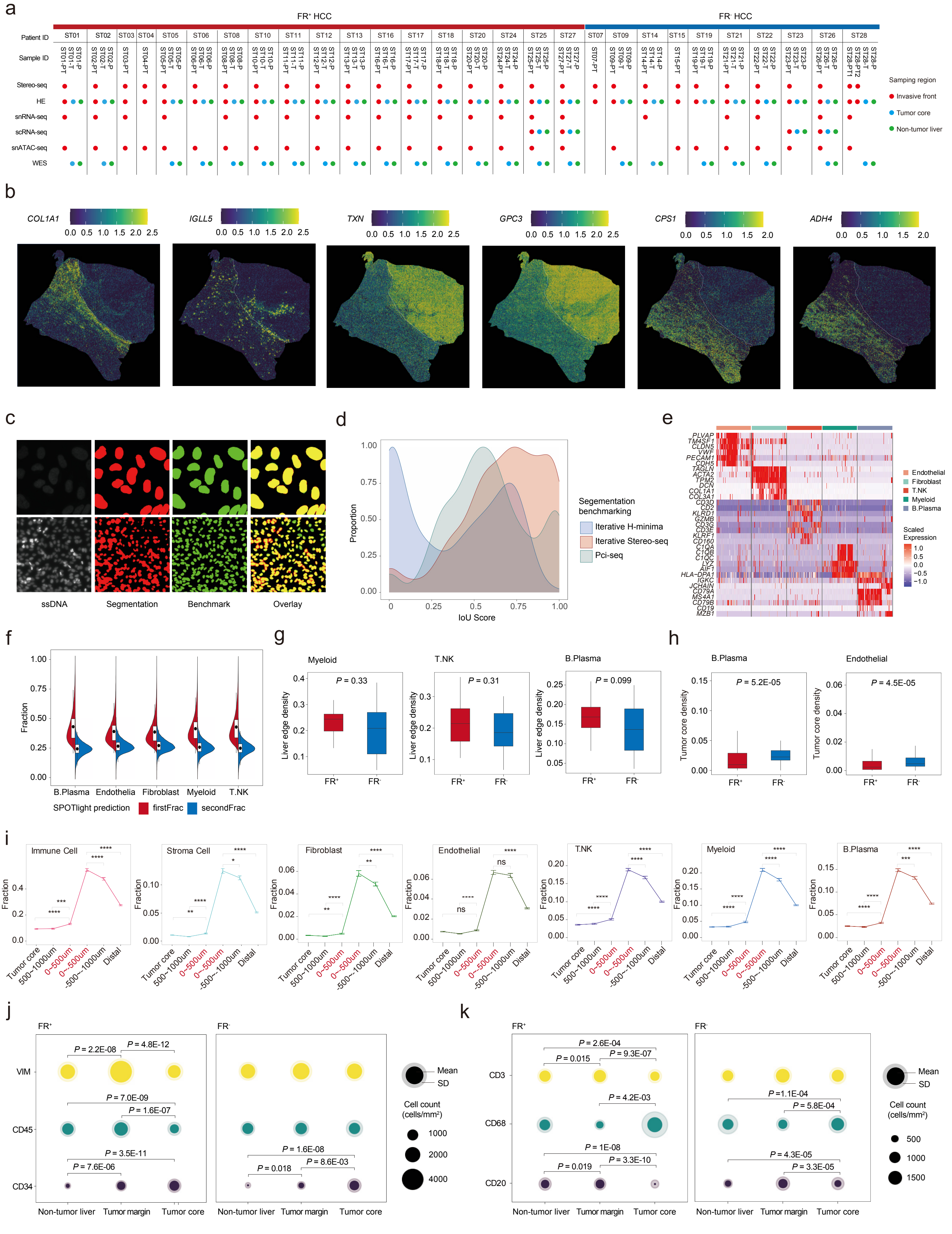

Supplementary Fig. S4

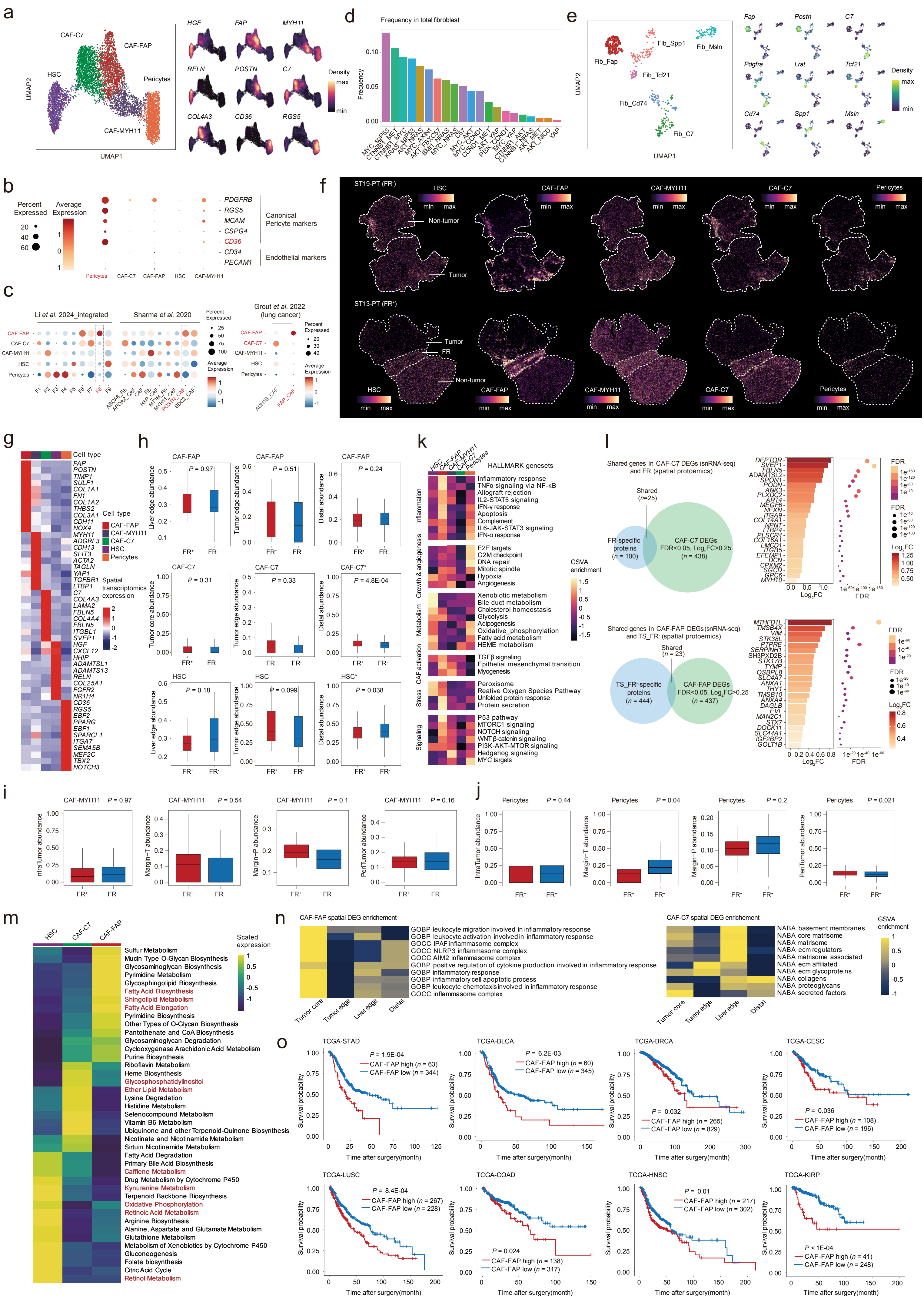

Supplementary Fig. S5

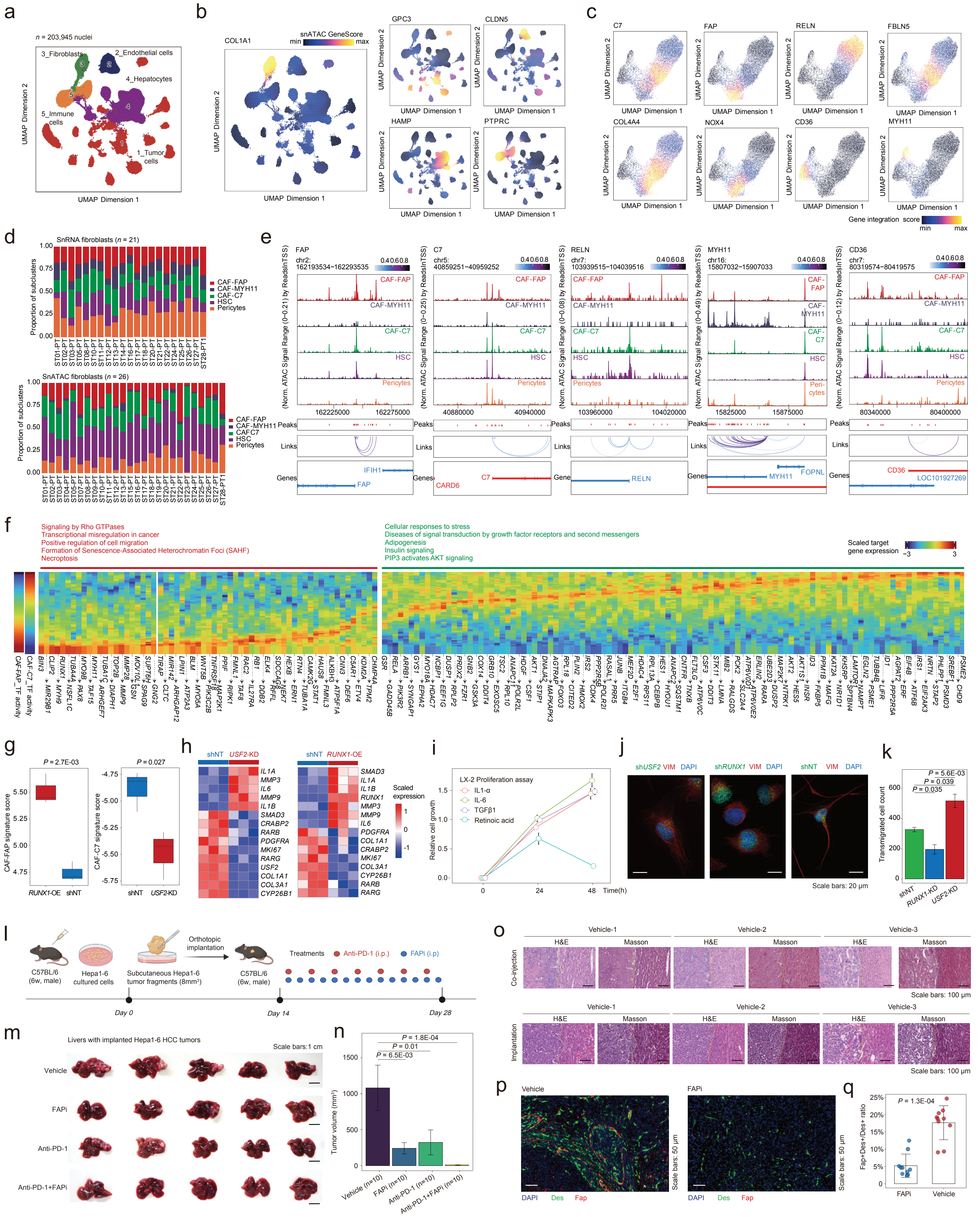

Supplementary Fig. S6

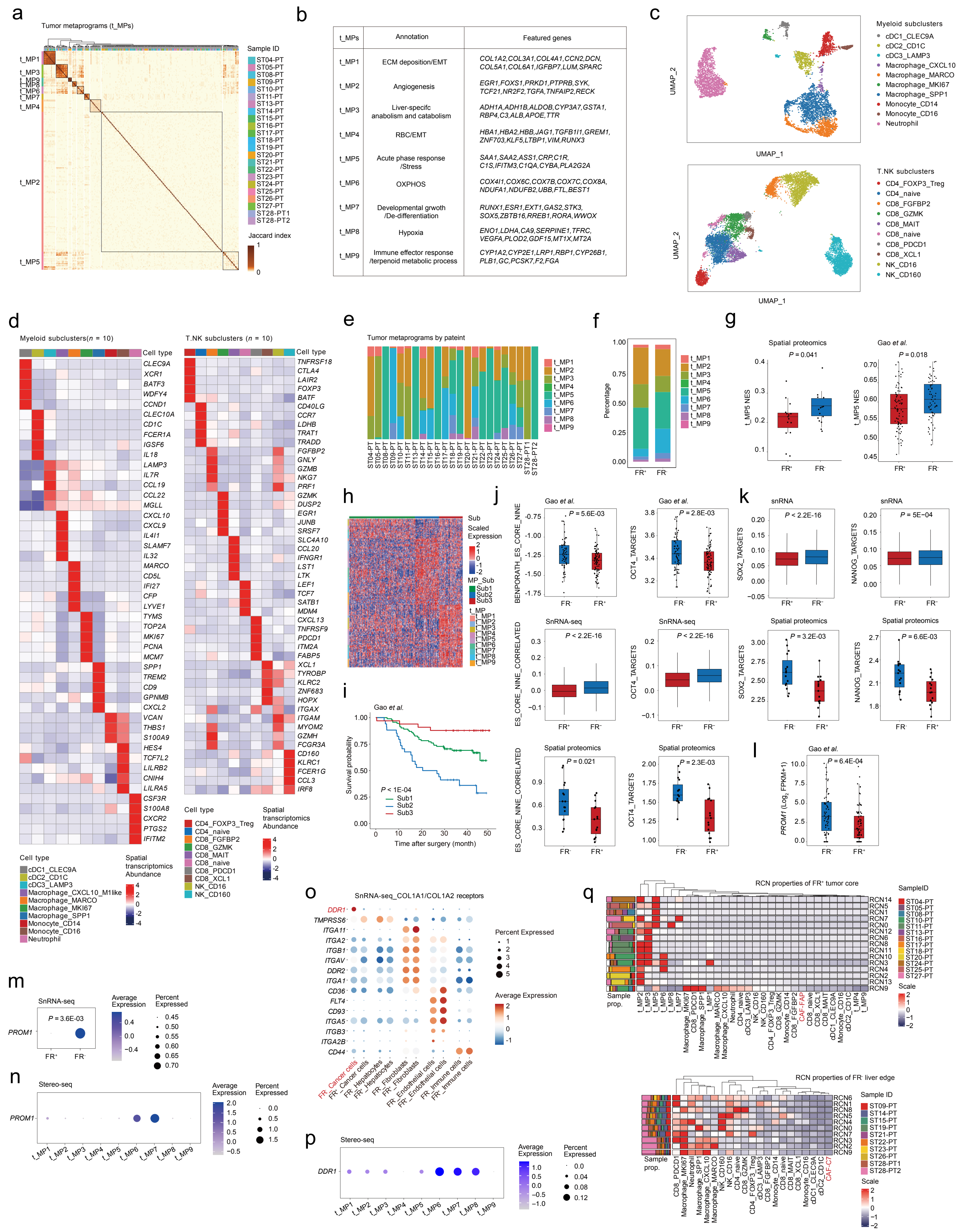

Supplementary Fig. S7

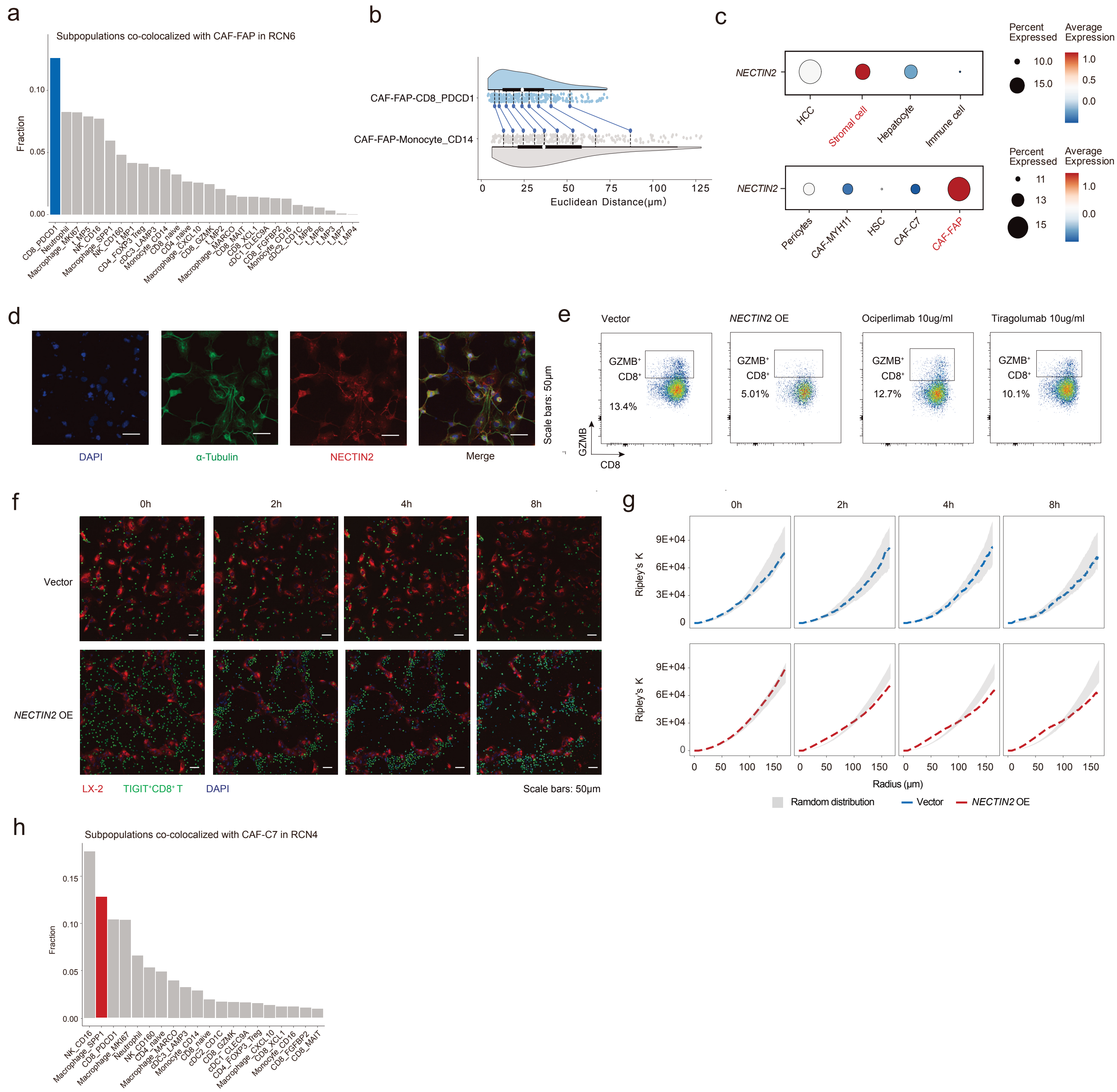

Supplementary Fig. S8

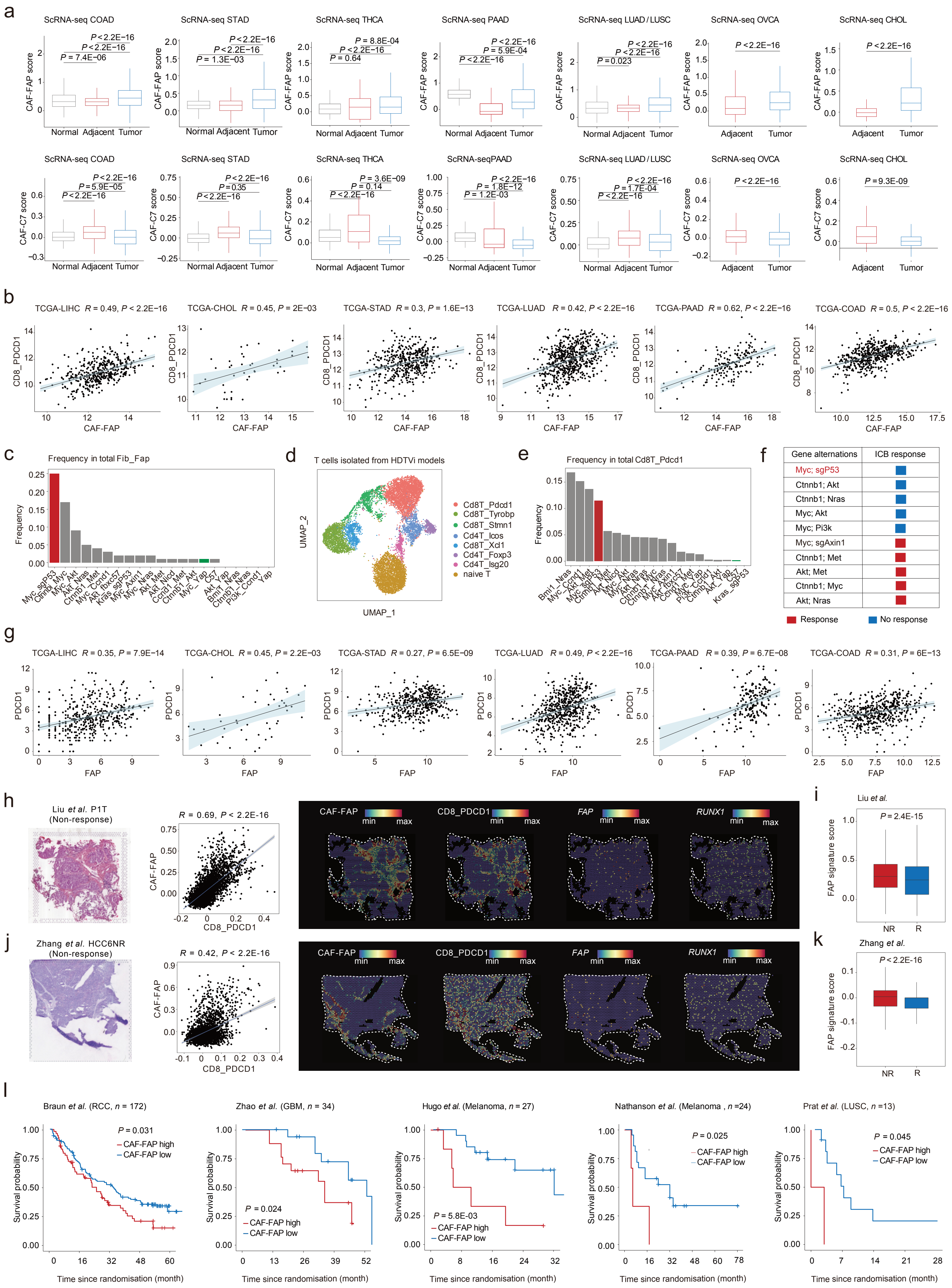

Supplementary figure legends

Supplementary Fig. S1. Morphological characteristics, clinical metrics, and molecular profiles regarding stromal architecture, Related to Fig. 1

a, b Representative H&E, Masson, Sirius Red, and IHC staining showing collagen fibers (Collagen I, Collagen IV), fibroblast activation (a-SMA), immune infiltration (CD3, CD8, CD68), tumor proliferation (Ki-67), and checkpoint molecules (PD-1, PD-L1) at the tumor core of FR^+ tumors (**a**) and FR^- tumors (**b**). Scale bars: 200 µm.

c Representative images of H&E (upper) and Masson (lower) showing the invasive boundary of primary liver cancer other than HCC and across liver metastases of 13 extra-hepatic origins. Scale bars: 100 µm.

d Forrest plot summarizing the multivariate analysis of factors associated with overall survival in cohort 1 (left) and cohort 2 (right).

e Bar plot showing the frequencies of FR^+ tumor in all samples stratified by tumor size in cohort 1 (upper) and cohort 2 (lower).

f Boxplot comparing the tumor purity in FR^+ and FR^- tumors. Wilcoxon test.

g Violin plot comparing the distribution of peritumor stromal content (upper) and peritumor immune infiltration (lower) predicted by deep learning in FR^+ and FR^- tumors. Wilcoxon test.

h Bar plot showing the frequencies of the histological grading of liver fibrosis in all samples according to their stromal architecture in cohort 1 and cohort 2. Chi-squared test.

i Bar plot showing tumor stromal architecture to the subgroups from the previously reported HCC molecular classifications.

j Functional enrichment of upregulated genes in FR^+ tumors versus FR^- tumors at the RNA level (left) and the protein level (right). Paired T-test.

k Boxplot comparing the XCELL-estimated levels of stroma components, fibroblasts, CD8 T cells, and CD8 naïve T cells between responders and non-responders in HCC tumor tissue from an external ICB cohort (EPR117672). Wilcoxon test.

l Boxplot comparing the response signatures generated from HCC ICB datasets. Wilcoxon test.

Supplementary Fig. S2. Proteomic profiling of stromal architecture, Related to Fig. 2

a Bar plot showing the total number of quantified proteins of different HCC micro-regions. All samples were collected by laser capture microdissection (LCM) and analyzed using 60-minute single-shot DIA runs.

b Venn diagrams of detected unique proteins by spatial proteomics compared with a previous proof-of-concept pipeline.

c Principal Component Analysis (PCA) comparing tumor stroma (TS) with tumor edge (TE) by spatial proteomics.

d Principal Component Analysis (PCA) comparing liver stroma (LS) with the

non-tumor liver edge (LE) by spatial proteomics.

e Expression of the ESTIMATE stromal signature across different spatial micro-regions. Kruskal-Wallis test.

f Heatmap of differential gene expression between micro-dissected FR^+ liver versus FR^- liver. Significantly enriched ontologies (adjusted *P* value < 0.05) by GSEA of the HALLMARK gene sets were listed on the right side of the heatmap.

g Heatmap of differential gene expression between micro-dissected tumor versus non-tumor liver. Significantly enriched ontologies (adjusted *P* value < 0.05) by GSEA of the HALLMARK gene sets were listed on the right side of the heatmap.

h Representative Masson trichrome staining (left) and SAA IHC staining (right) of FR^+ tumors and FR^- tumors. Scale bars: 200 µm.

i Representative Masson trichrome staining (left) and IHC staining showing the spatial distribution of stress and inflammatory proteins (CRP, HSP90, MMP14, IL-8) at FR^- tumor core (upper) and FR^+ tumor margin (lower). Scale bars: 400 µm.

j Expression of the GO_BP_WOUND_HEALING signature across 5 stromal micro-regions. Wilcoxon test.

Supplementary Fig. S3. Single-cell spatial transcriptomics of major clusters, Related to Fig. 3

a Data overview of the spatial multi-omics study cohort. Samples are organized by their stromal architecture. Dots colored according to sampling region indicate data availability.

b Spatial visualization of marker gene expression representing the 3 major regions revealed by spatially confined clustering: *COL1A1*, *IGLL5* for stroma, *TXN*, *GPC3* for HCC tumor, and *CPS1*, *ADH4* for the non-tumor liver.

c Representative images illustrating the results of applying the iterative cell segmentation algorithm to the 2018 Data Science Bowl dataset in both relatively dim (top) and bright (bottom) ssDNA images. Overlaid masks (colored in yellow) show high consistency between our segmentation (colored in red) and manually annotated ground truth (colored in green).

d Intersection over Union (IoU) curve comparing iterative cell segmentation algorithm to published segmentation methods in sensitivity and specificity for nuclei detection.

e Heatmap showing top DEGs of the 5 major clusters annotated by scRNA-seq.

f Grouped violin plots showing the distribution of the primary cell type and the secondary cell type.

g, h Boxplot comparing the spatial enrichment of remaining major clusters in defined spatial zones of the liver edge (**g**) and the tumor core (**h**) in FR^+ tumors versus FR^- tumors. Wilcoxon test.

i Line plot showing the average fraction of total immune or stroma cell and 5 major cell clusters among all cell components (including parenchymal cells) in different layers around the border of HCC. T-test.

j, k Comparisons of major TME cell distribution among different spatial regions inside FR^+ tumor (left) or FR^- tumor (right). Circle plots representing the mean and standard deviation (SD) of the cell densities (cells/mm^2) in Tissue Microarrays (TMAs) of the paired non-tumor liver, tumor margin, and tumor core from cohort 2 (n =159; FR^+, n = 92; FR^- n = 67). The statistics of fibroblasts (VIM^+), endothelial cells ($CD34^+$), and immune cells ($CD45^+$) were included in (**j**), while T cells ($CD3^+$), macrophages ($CD68^+$), and B cells ($CD20^+$) were displayed in (**k**). Wilcoxon test.

Supplementary Fig. S4. Fibroblast transcriptional programs in human and murine HCCs, Related to Fig. 4

a UMAP representation of fibroblast subsets (left) and density estimation of fibroblast marker gene expression (right) by snRNA-seq of ST-paired tissue.

b Dot plot showing the expression of canonical pericyte markers, *CD36* and endothelial markers across fibroblast subclusters.

c Dot plot showing the expression of fibroblast signatures from published HCC datasets (left) and signatures defined by Grout *et al.* in human lung cancer (right).

d Bar plot showing the frequencies of fibroblasts taken up by different tumorigenic genotypes induced by hydrodynamic tail vein injection (HDTVi).

e UMAP representation of fibroblast subsets (left) and density estimation of fibroblast marker gene expression (right) from an original scRNA-seq dataset of mouse HDTVi HCC tumors.

f Spatial visualization of the marker gene expression of each fibroblast subset ($Log_2FC>1$) in FR^- (upper) and FR^+ tumors (lower).

g Heatmap showing the marker genes expression of the 5 fibroblast subsets by single-cell spatial transcriptomics.

h Boxplot comparing the spatial enrichment of CAF-FAP (top), CAF-C7 (middle), and HSC (bottom) in defined spatial zones in FR^+ tumors versus FR^- tumors. Wilcoxon test.

i, j Boxplot comparing the spatial enrichment of CAF-MYH11 (**i**) and pericytes (**j**) in defined spatial zones in FR^+ tumors versus FR^- tumors. Student's T-test.

k Heatmap showing the HALLMARK gene sets that were upregulated in each fibroblast subcluster by GSVA analysis.

l Shared upregulated genes between CAF-C7 (snRNA-seq) and FR (spatial proteomics) (upper), and between CAF-FAP (snRNA-seq) and TS_FR^- (spatial proteomics) (lower), showing the differential expression of these shared genes in fibroblast snRNA-seq data. The FDR values are depicted in Log_{10} scale.

m Heatmap showing the upregulated metabolic pathways in CAF-FAP, CAF-C7, and HSC by GSVA analysis.

n Heatmap showing the upregulated featured pathways along the tumor-margin-liver axis of CAF-FAP (inflammatory response) and CAF-C7 (ECM organization) by GSVA analysis.
o Kaplan-Meier curves for overall survival across 8 different cancer types from the TCGA atlas based on CAF-FAP signature. Log-rank test.

Supplementary Fig. S5. Epigenetic regulation of fibroblast heterogeneity, Related to Fig. 5
a UMAP projection of integrated snRNA-seq and snATAC-seq of total cells colored by major cell clusters.
b, c UMAP projection of major TME cell clusters (**b**) and fibroblast subsets (**c**) colored by gene activity scores reflecting the binding accessibility of their marker genes.
d Bar plot showing the compositions of fibroblast across samples by snRNA-seq (top) and by snATAc-seq (bottom).
e Genomic tracks for accessibility around fibroblast marker genes for corresponding subclusters. Peaks called in the integrated scATAC-seq data and peaks-to-gene links are shown below the tracks. Marker peaks for each fibroblast subtype are listed below the tracks.
f Pseudotime heatmap showing the enriched pathways and target genes of CAF-FAP and CAF-C7 TF activities.
g Boxplot showing the expression of CAF-FAP score in *RUNX1*-OE LX-2 cells versus control (left) and CAF-C7 score in *USF2*-KD LX-2 cells versus control (right). Student's t-test.
h Heatmap displaying genes related to inflammation, proteolysis, TGF-β signaling, and retinoid metabolism in *USF2*-KD (left) or *RUNX1*-OE (right) versus control (shNT) in LX-2 cells.
i Relative cell growth of LX-2 cells stimulated by 10ug/mL human IL-1α, IL-6, TGF-β1, and retinoic acid in vitro.
j Representative images of control (shNT), *RUNX1*-KD (shRUNX1), and *USF2*-KD (sh*USF2*) LX-2 cells with vimentin (VIM) staining. Scale bars: 20 µm.
k Transwell migrated cell count of *USF2*-KD (*n* = 3), *RUNX1*-KD (*n* = 3), and control (*n* = 3) LX-2 cells after 24 hours from DMEM/F12 towards DMEM with 20%FBS. Wilcoxon test.
l Schematic of orthotopic HCC implantation and drug interventions.
m Representative images of harvested livers from orthotopic HCC models receiving different treatments (vehicle *n* = 10, FAPi *n* = 10, Anti-PD-1 *n* = 10, Anti-PD-1+ FAPi *n* = 10). Scale bars: 1cm.
n Boxplots showing the tumor volume by different regimens (FAPi *n* = 10, Anti-PD-1 *n* = 10, Anti-PD-1+ FAPi *n* = 10) and control (vehicle *n* = 10). Wilcoxon test.
o Representative H&E and Masson trichome staining images showing the absence of FR at tumor margin of in the Hepa1-6 orthotopic models. Upper,

Hepa1-6-mHSC co-injection. Lower, tumor block implantation. Scale bars: 100 μm.

p Multi-plex imaging showing Des and Fap protein expression in mouse Hepa1-6 orthotropic models treated by vehicle (left) and FAPi (right).

q Boxplot illustrating the frequencies of CAF-FAP relative to total CAF in representative areas of the vehicle group compared to the FAPi group. Wilcoxon test.

Supplementary Fig. S6. Malignant and immune compartments coordinated with CAF-FAP and CAF-C7, Related to Fig. 6

a Hierarchical clustering of pairwise Jaccard similarities between 9 NMF tumor metaprograms (t_MPs) identified across malignant cells from single-cell spatial transcriptomics.

b Annotation and top feature genes from each t_MP (t_MP1 to t_MP9).

c UMAP representation of myeloid subsets (upper) and T and NK subsets (lower) by scRNA-seq.

d Heatmap showing the marker genes expression of the myeloid subclusters (left) and T and NK subclusters (right) by single-cell spatial transcriptomics.

e Bar plot showing the frequency of t_MPs in all tumor margin samples.

f Bar plot displaying the composition of t_MPs in FR^+ tumors and FR^- tumors.

g Boxplot comparing the expression of t_MP5 features in FR^+ tumors versus FR^- tumors using the spatial proteomics data (left) and RNA-seq data in cohort 2 (right). Wilcoxon test.

h Heatmap showing patients in cohort 2 were further classified into 3 MPsubs according to the expression of the top 50 feature genes in each t_MP.

i Kaplan-Meier plot showing overall survival based on the MP_sub classification in cohort 2. Log-rank test.

j, k Boxplot illustrating the significant upregulation of stem-cell-like gene sets in FR^- tumors. Wilcoxon test.

l, m Boxplot and dot plot comparing *PROM1* expression between FR^+ and FR^- samples in Gao *et al.* (**l**) and snRNA-seq(**m**), respectively. Wilcoxon test.

n Dot plot comparing *PROM1* expression across 9 tumor metaprograms.

o Dot plot showing the gene expression of COL1A1 and COL1A2 receptors among major TME cell types split by the FR^+ and FR^- groups.

p Dot plot comparing *DDR1* expression across 9 tumor metaprograms.

q RCN properties at the tumor core of FR^+ tumors (upper) and at the liver edge of FR^- tumors (lower). Heatmap shows the abundance of cell types with each RCN, while bar plots to the left of the heatmap represent the distribution of patients with each cluster.

Supplementary Fig. S7. Analysis and validation related to cell-cell interactions in stromal hubs, Related to Fig. 6

a Bar plots showing ranked fractions of cell types co-localized with CAF-FAP in RCN6.
b Shift plots representing the CAF-FAP-CD8_PDCD1 distance and CAF-FAP-Monocyte_CD14 distance in the intratumor zone of FR$^-$ tumors. Significance is calculated for percentiles of 10, 20, 30, 40, 50, 60, 70, 80, 90 by the Robust Harrell-Davis quantile estimator. The blue line represents a significant difference ($P < 0.05$, less CAF-FAP-CD8_PDCD1 distance), and the grey line represents non-significance for the percentile.
c Dot plot showing the gene expression of *NECTIN2* among major TME cell types (upper) and fibroblast subpopulations (lower).
d Representative images of immunocytochemistry showing the expression of NECTIN2 on *NECTIN2*-OE LX-2 cells. Scale bars: 50 µm.
e Representative images of flow cytometry showing the proportions of GZMB$^+$CD8$^+$ T cells for the *NECTIN2*-OE LX-2 cells, vector, and TIGIT inhibitors (Ociperlimab and Tiragolumab) by co-culture.
f High-content cell imaging showing the difference between the TIGIT$^+$ CD8$^+$ T cell (green) interaction with *NECTIN2*-OE LX-2 cells and control (red) at different time points in vitro. Scale bars: 50 µm.
g Line plot of the Ripley's K-Function evaluating the aggregation of TIGIT$^+$ CD8$^+$ T cells at different time points in vitro for the *NECTIN2*-OE LX-2 cells and control.
h Bar plots showing ranked fractions of cell types co-localized with CAF-C7 in RCN4.

Supplementary Fig. S8. Extended validation of stromal hubs across cancer types and upon immunotherapy treatment, Related to Fig. 7
a Boxplots showing the difference in CAF-FAP and CAF-C7 scores from different spatial locations (tumor, adjacent, normal) by different cancer types. Wilcoxon test.
b Scatter plots showing the correlation of CAF-FAP and CD8_PDCD1 abundance. Pearson's correlation.
c Bar plot showing the frequencies of Fib_fap taken up by different tumorigenic genotypes induced by HDTVi.
d UMAP representation of T cell subsets in HDTVi HCC models, largely repopulating human HCC T-cell subpopulations.
e Bar plot showing the frequencies of Cd8T_Pdcd1 taken up by different tumorigenic genotypes induced by HDTVi.
f Table summarizing the immunotherapy response corresponding to genetic alternations in HDTVi HCC models.
g Scatter plots showing the correlation of *FAP* and *PDCD1* gene expression. Pearson's correlation.
h Spatial characterization of the inflammatory hubs in HCC tumors that did not respond to immunotherapy in the dataset of Liu *et al.*, showing paired histology, spatial distribution, and correlation of CAF-FAP and CD8_PDCD1 and marker gene expression. Pearson correlation.

i Boxplot showing the CAF-FAP signature across stroma regions of non-responders (NR) versus responders(R) in the *Liu et al*. dataset. Wilcoxon test.
j Spatial characterization of the inflammatory hubs in HCC tumors that did not respond to immunotherapy in the Zhang *et al.* dataset, showing paired histology, spatial distribution, and correlation of CAF-FAP and CD8_PDCD1 and marker gene expression. Pearson correlation.
k Boxplot showing the CAF-FAP signature across stroma regions of non-responders (NR) versus responders (R) in the Zhang *et al.* dataset. Wilcoxon test.
l Kaplan-Meier plot depicting overall survival stratified by the CAF-FAP signature in various ICB cohorts. Log-rank test.
